# Reconstructing the ups and downs of primate brain evolution: implications for adaptive hypotheses and *Homo floresiensis*

**DOI:** 10.1186/1741-7007-8-9

**Published:** 2010-01-27

**Authors:** Stephen H Montgomery, Isabella Capellini, Robert A Barton, Nicholas I Mundy

**Affiliations:** 1 Department of Zoology, University of Cambridge, Downing Street, Cambridge, UK; 2 Evolutionary Anthropology Research Group, Department of Anthropology, Durham University, Dawson Building, South Road, Durham, DH1 3LE, UK

## Abstract

**Background:**

Brain size is a key adaptive trait. It is often assumed that increasing brain size was a general evolutionary trend in primates, yet recent fossil discoveries have documented brain size decreases in some lineages, raising the question of how general a trend there was for brains to increase in mass over evolutionary time. We present the first systematic phylogenetic analysis designed to answer this question.

**Results:**

We performed ancestral state reconstructions of three traits (absolute brain mass, absolute body mass, relative brain mass) using 37 extant and 23 extinct primate species and three approaches to ancestral state reconstruction: parsimony, maximum likelihood and Bayesian Markov-chain Monte Carlo. Both absolute and relative brain mass generally increased over evolutionary time, but body mass did not. Nevertheless both absolute and relative brain mass decreased along several branches. Applying these results to the contentious case of *Homo floresiensis*, we find a number of scenarios under which the proposed evolution of *Homo floresiensis' *small brain appears to be consistent with patterns observed along other lineages, dependent on body mass and phylogenetic position.

**Conclusions:**

Our results confirm that brain expansion began early in primate evolution and show that increases occurred in all major clades. Only in terms of an increase in absolute mass does the human lineage appear particularly striking, with both the rate of proportional change in mass and relative brain size having episodes of greater expansion elsewhere on the primate phylogeny. However, decreases in brain mass also occurred along branches in all major clades, and we conclude that, while selection has acted to enlarge primate brains, in some lineages this trend has been reversed. Further analyses of the phylogenetic position of *Homo floresiensis *and better body mass estimates are required to confirm the plausibility of the evolution of its small brain mass. We find that for our dataset the Bayesian analysis for ancestral state reconstruction is least affected by inclusion of fossil data suggesting that this approach might be preferable for future studies on other taxa with a poor fossil record.

## Background

Phylogenetic comparative methods and ancestral state reconstruction play important roles in evolutionary biology. They enable historical evolutionary processes, and the function and evolution of specific traits, to be inferred from patterns of diversity in extant species [1-3]. Extant primate brains, which vary from 1.8 g (*Microcebus murinus*) to 1330 g (*Homo sapiens*), fall within the range of non-primate mammalian brain masses [4]. However, after correcting for allometric scaling with body mass, primates have relatively large brains compared to most other mammals [5]. A trend towards brain expansion is assumed to have occurred throughout primate evolution [6] and this has been interpreted as an indication of directional selection on cognitive abilities, due, for example, to arms races in social cognition [7,8].

Recent studies, however, indicate that brain size, measured either in volume or mass, may have decreased in some vertebrate lineages [9,10]. Decreases in both absolute and relative brain size appear to have occurred in a number of taxa including birds [11], bats [10], bovids [12], elephants [13] and hippopotami [14,15]. Dwarfism following island isolation (*the island rule*) can account for some of these decreases [15,16] but not all. For at least some of these cases it is likely that a reduction in brain size has occurred to meet the demands of the species' changing ecological needs rather than being due to geographical isolation *per se *[10,11].

Although many studies have investigated the possible selective advantages and disadvantages of increased brain size in primates [5,17-21], few consider how frequently brain size has reduced. Periods of primate evolution which show decreases in brain size are of great interest as they may yield insights into the selective pressures and developmental constraints acting on brain size. Bauchot & Stephan [22] noted the evolution of reduced brain size in the dwarf Old World monkey *Miopithecus talapoin *and Martin [23] suggested relative brain size in great apes may have undergone a reduction based on the cranial capacity of the extinct hominoid *Proconsul africanus*. Taylor & van Schaik [24]reported a reduced cranial capacity in *Pongo pygmaeus morio *compared to other Orang-utan populations and hypothesise this reduction is selected for as a result of scarcity of food. Finally, Henneberg [25] has shown that during the late Pleistocene human absolute brain size has decreased by 10%, accompanied by a parallel decrease in body size.

**Figure 1:**
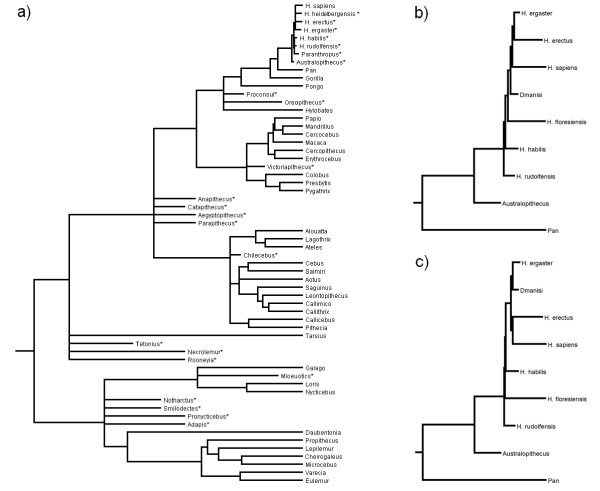
**Phylogeny of primates with extinct primates**. a) Phylogeny used for main reconstruction analysis. Extinct primates are denoted with an asterisk (*); b) and c) Phylogenies off *Homini *used for the *H. floresiensis *analysis based on the two most parsimonious topologies from Argue *et al*. [43], the rest of the phylogeny was left as shown in a). b) corresponds to Argue *et al*.'s Tree 1 and c) to Tree 2. Branches are drawn proportional to time. This figure was prepared in Mesquite [110].

The importance of understanding the evolution of reduced brain size in primates has recently been brought into sharp focus with the discovery of a small-brained hominin, *Homo floresiensis*, which overlapped both geographically and temporally with modern humans [26,27]. This has challenged our understanding of human evolution and created much debate about whether *H. floresiensis *was a distinct species or a pathological example of modern humans [28-30]. Studies describing the endocast and post-cranial features of the type specimen (LB1) have resulted in mixed conclusions [31-38]. Analyses using known cases of dwarfism to model brain and body size reduction in *H. floresiensis *from an ancestral *Homo erectus *population suggested insular dwarfism cannot explain the smaller brain and body size [[39,40]; but also [15]]. However, recent studies have found that both the degree and temporal rate of reduction in brain and body size observed in *H. floresiensis*, assuming ancestry with *H. erectus*, fall within the range of size reductions in other island primate species [41,42]. An alternative phylogenetic hypothesis for *H. floresiensis *has recently been proposed and indicates that this species may not have evolved by insular dwarfism of a known *Homo *species [43]. Instead Argue et al [43] propose two equally parsimonious cladograms in which *H. floresiensis *is a distinct early species *Homo*, emerged after *H. rudolfensis *and either before or after *H. habilis *(Figure 1b &1c). The debate about the place of *H. floresiensis *in the primate tree and the possible evolutionary significance of its small size and encephalization could be illuminated by placing the specimens in the context of a broader phylogenetic analysis.

Reconstructing ancestral brain and body sizes provides a means of testing the generality of the trend of increasing brain and body size through primate evolution. It also provides estimates of brain and body sizes at key points along the primate phylogeny allowing inferences to be made about the ecology of the ancestors of key clades, based on what we know about the relationship between body size, ecology and life history traits in living primates [44,45]. However, before making any inferences based on estimated ancestral states it is vital to perform a thorough comparison of reconstruction methods in order to obtain the most reliable estimates. Previous studies have used weighted square change parsimony and maximum likelihood (ML) to reconstruct ancestral character states and infer the adaptive origins of phenotypes [46-49] and to study important genotype-phenotype associations [50]. However, basing conclusions on these methods may be problematic as they fail to provide reliable estimates when there are directional evolutionary changes and can also be adversely affected when ancestral states fall outside the range of extant species [51,52], as is expected to be the case for primate brain evolution [6]. Incorporating data from fossils can improve ancestral reconstruction estimates as they may more completely describe the range and temporal distribution of the character's history, and thus help improve the estimated nodal values both in the presence of directional trends and when ancestral values are markedly different from extant species values [47,53]. More recently, a method to model directional change and find the best-fitting models of evolution *prior *to ancestral state reconstruction in Bayesian framework has been developed [54,55]. Whether this method performs better than parsimony and ML in estimating ancestral states and whether it is influenced by including fossil data like other methods has yet to be examined using real datasets.

Here we investigate the evolution of brain and body mass in Primates and assess whether brain mass and body mass show evidence of directional trends. Given the strong allometric relationship between brain and body mass [5,6], relative brain size is most commonly used in comparative studies [56] that aim to test the evolutionary significance of an increased brain mass above that predicted from a species' body mass [for example [7,57]]. However, absolute brain mass is of evolutionary relevance too [58,59]; it may be related to cognitive ability [60] and it is correlated with neuron number [61] which in turn is likely to have important implications for cognitive performance [4]. Furthermore, analysis of absolute brain and body size is necessary in order to interpret the nature of evolutionary changes in relative brain size. We therefore investigated the evolutionary history of both relative and absolute brain size.

First, we adopted three approaches to reconstructing the evolutionary history of these traits: weighted squared-change parsimony, maximum likelihood (ML) and Bayesian Markov-chain Monte Carlo (MCMC) [54,55,62,63] (see Methods), and performed each analysis with and without inclusion of fossil data. Following previous studies showing that ancestral state reconstruction is improved by including fossil data particularly when traits evolved under a directional trend [47,53], we assessed the sensitivity of each method to the inclusion of fossil data, and also compared estimates across methods. The aim of the model comparison is to test whether any method produces consistent estimates with and without the inclusion of fossils, as this might suggest a more robust method which is less affected by aspects of the trait's evolution which decrease the accuracy of ancestral state reconstructions, such as directional trends or ancestral values which lie outside of the range of extant species. Second, to explicitly model and assess statistically whether there was a directional increase in brain and body mass, we compared ancestral reconstruction when a directional constant-variance random walk model of evolution was assumed versus a non-directional constant-variance random walk model as implemented in BayesTraits [54,55], using the phylogeny with fossil species included. We then examined the pattern of change in brain and body size through the tree under the most supported model.

We discuss the implications of our results for hypotheses on the adaptive origins of modern primates, and identify branches along which brain mass has increased greatly or at a high rate, or along which brain mass has decreased in either absolute or relative terms. Finally we use our results to evaluate alternative scenarios about the origin of *H. floresiensis*, specifically from three different populations of *H. erectus *and from *H. habilis*. This analysis aims to evaluate whether descent of *H. floresiensis *from a putative ancestral population involves a decrease in brain and body mass that is beyond those observed in other primate lineages. Our analyses show that ancestral state reconstruction can be an informative way to infer evolutionary processes using data from living species, but highlight the need to assess the reliability of these estimates when doing so.

## Results and discussion

### Ancestral reconstructions: congruence between estimates with and without fossil data

**Figure 2:**
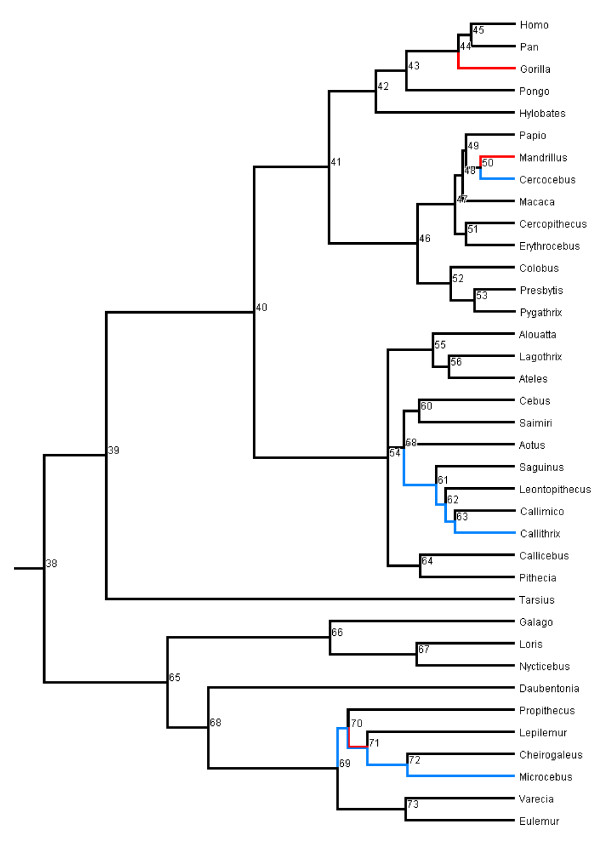
**Phylogeny of extant primate genera**. Branches are drawn proportional to time. This figure was prepared in Mesquite [110].

Previous studies have used either volume or mass as a measure of brain size. Here we used log_10_-transformed brain and body mass in all analyses. Brain and body mass estimates were collected for 37 extant and 23 extinct primate species (Additional file 1, Table S1). We first performed the reconstruction analysis using three alternative approaches (parsimony, ML and Bayesian MCMC in BayesTraits). Following Webster & Purvis [64], we then assessed the reliability of the estimates, by comparing results obtained with and without fossil data using correlations. In comparing results, estimates of ancestral states for each node obtained in the Bayesian framework were taken as the average of the posterior distribution. The phylogenies that we used are shown in Figure 1 (extant and extinct species) and Figure 2 (extant species).

**Figure 3:**
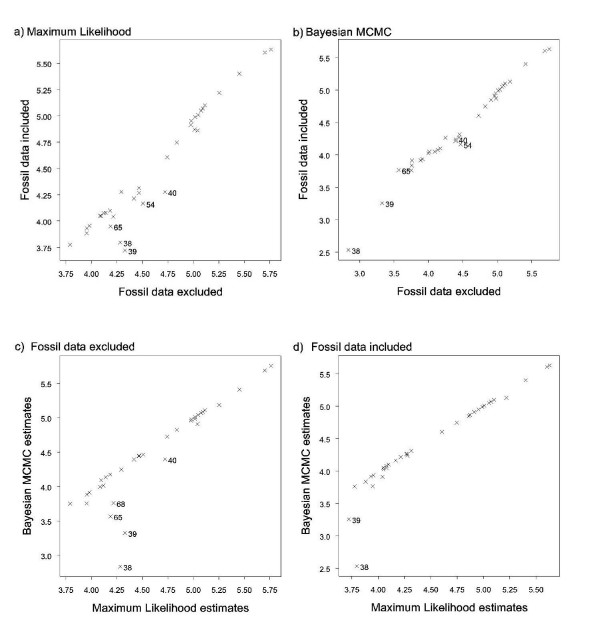
**Correlations between estimates of absolute brain mass in log(grams)**. **a) **Correlations are shown with and without fossil data using ML; **b) **with and without fossil data using Bayesian MCMC; **c) **without fossil data between ML and Bayesian MCMC results; **d) **with fossil data between ML and bayesian MCMC results. Numbers indicate nodes in figure 2.

Ancestral values for all nodes of the tree with and without the inclusion of fossil data were highly correlated for absolute brain mass (parsimony, Spearman's correlation coefficient (*r*_*s*_) = 0.932; ML, *r*_*s *_= 0.932; Bayesian MCMC, *r*_*s *_= 0.993, all *P *< 0.001) and body mass (parsimony, *r*_*s *_= 0.939; ML, *r*_*s *_= 0.941; Bayesian MCMC, *r*_*s *_= 0.960, all *P *< 0.001). As expected, ML and parsimony methods produce almost identical results for estimates made with (*r*_*s *_= 1.000, *P *< 0.001) and without fossils (*r*_*s *_= 1.000, *P *< 0.001). We therefore only present the results of further comparisons between ML and Bayesian MCMC. The lower *r*_*s *_values in the parsimony and ML analyses are caused by increased disparity between the estimates at deeper nodes. In particular, estimates of log(brain mass) using fossils are 10-15% lower for the root (Figure 2, node 38), the ancestral haplorhine (39), the ancestral anthropoid (40) and the ancestral New World monkey (54) than estimates made without inclusion of the fossil data (Figure 3a), suggesting the accuracy of the estimates decreases at the deeper nodes when fossil data are not used. The standard errors in ML analysis support this conclusion, being larger for deeper nodes. Conversely, the results of the Bayesian MCMC analysis do not show this disparity and deep nodes fall on the same line as shallower nodes (Figure 3a &3b) although confidence intervals of the root estimate are still higher than those of all other nodes (Additional file 1, Table S3).

The results from the Bayesian analyses agree more strongly with ML when fossil data are included than when they are excluded for both brain mass (with fossils: *r*_*s *_= 0.995, *P *< 0.001; without fossils: *r*_*s *_= 0.923, *P *< 0.001; Figure 3c and 3d) and body mass (*r*_*s *_= 0.981, *P *< 0.001 with fossils; *r*_*s *_= 0.926, *P *< 0.001 without fossils). The greatest disparity between estimates made without fossils between parsimony/likelihood analysis and Bayesian MCMC analysis are found at the root (node 38), the ancestral haplorhine (39), the ancestral anthropoid (40), the ancestral strepsirhine (65) and the ancestral lemur (68). When fossil data are included disparity between the MCMC & ML remain for nodes 38 and 39, with the estimates from the Bayesian analysis being lower than those made by the other methods.

To measure relative brain mass for the species in the tree we performed a phylogenetically controlled GLS regression analysis between log(brain mass) and log(body mass) using ML in BayesTraits (see Methods), that returned the following fit line: log(brain mass) = 2.18 + 0.684 [log(body mass)]. We then reconstructed ancestral character states for relative brain size with two alternative approaches. With the first approach, which we term *residuals second*, we first inferred ancestral brain and body sizes at each node and then derived relative brain size as the residual brain size on body size using the ancestral state estimates at the nodes and the phylogenetically controlled GLS equation. This approach has the advantage of first finding the best fitting model for brain and body mass, but does not explicitly model the correlated evolution of brain and body mass, and cannot be used for testing directionality in the evolution of encephalization (relative brain mass) if brain and body size evolved under different models. With the second approach, *residuals first*, we explicitly modelled the evolution of encephalization as relative brain size, by first calculating the residuals of brain on body mass in the extant species with the phylogenetically controlled fit line, and then used these residuals as data to perform an ancestral state reconstruction of relative brain size. This approach has the advantage of modelling encephalization but it cannot incorporate two distinct models for brain and body mass should they evolve under different models. These two approaches, however, produced very similar relative brain size values, as the residuals at each node returned by the two methods were highly correlated (*r*_*s *_= 0.979, *P *< 0.001) and we therefore present only the results of the *residuals second *method.

**Figure 4:**
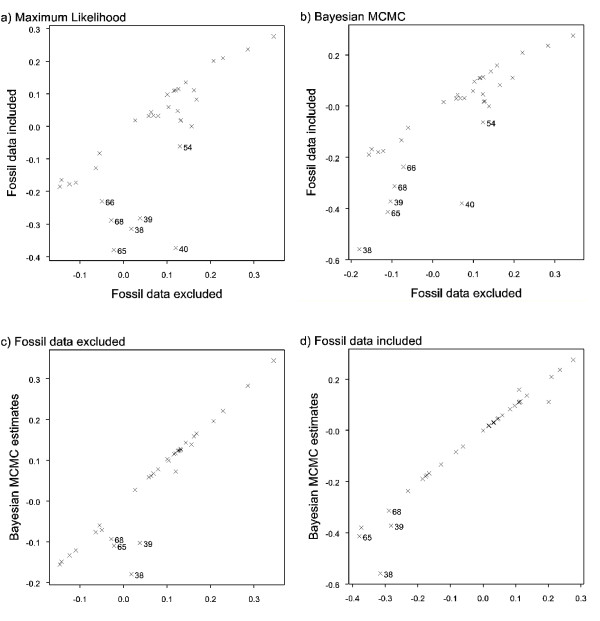
**Correlations between estimates of relative brain mass**. **a) **Correlations are shown with and without fossil data using ML; **b) **with and without fossil data using Bayesian MCMC; **c) **without fossil data between ML and Bayesian MCMC results; **d) **with fossil data between ML and bayesian MCMC results. Numbers indicate nodes in Figure 2.

The level of congruence between ancestral state estimates made with and without fossil data was much lower for relative brain mass than for absolute brain mass. Spearman's rank correlations for all three methods were highly significant although *r*_*s *_values were much lower for ML (*r*_*s *_= 0.743, *P *< 0.001) and parsimony (*r*_*s *_= 0.743, *P *< 0.001) than for the Bayesian analysis (*r*_*s *_= 0.835, *P *< 0.001). All three approaches performed poorly when estimating ancestral states of deep nodes, as indicated by the large disparities between estimates made with and without fossils (Figure 4a and 4b). Estimates of ancestral relative brain mass using parsimony and ML were highly consistent both with and without fossil data (for both analyses: *r*_*s *_= 1.000, *P *< 0.001). The results from Bayesian MCMC analysis were again more similar to those estimated by the two other methods when fossil data were included (*r*_*s *_= 0.994 with fossils, *r*_*s *_= 0.968 without; Figure 4c and 4d).

It is interesting to note that for all three approaches the estimated brain mass of the last common ancestor of humans and chimpanzees is larger when fossil data are not included. For example, the average estimate of brain mass for the *Homo-Pan *ancestor using MCMC analysis was 569.4 g (95% CI: 567.7 - 572.2 g) without fossils, while when fossils were included the average estimate was 425.6 g (95% CI: 424.2 to 426.8 g). This suggests the mass of the human brain can exert a large influence over ancestral state reconstructions in the great ape clade.

To summarise, parsimony and ML produced ancestral state estimates that were more discrepant between analyses with and without fossils when compared to estimates obtained with Organ *et al*.'s [55] method in a Bayesian framework. Moreover, the estimates of the Bayesian analysis were more consistent with those produced by ML and parsimony when fossil data were included. These results thus suggest that Organ *et al*.'s [55] method is more robust and therefore preferable for reconstructing ancestral states with our dataset. Although we cannot say whether this approach will generally perform most reliably when reconstructing ancestral states in taxa where little or no fossil data are available, our results suggest that this might be the case. It would thus be interesting to test whether Organ *et al*.'s [55] method for reconstructing ancestral states does perform better than parsimony and ML in the absence of fossil data using simulations and datasets where the ancestral states are known [for example, [51]].

One possible reason for lower consistency in estimates with and without fossils, particularly for ML and parsimony, might be the presence of directional trends [51,64]. Although this problem can in part be mitigated by incorporating fossil data that provide temporal information and variation not observed among living species and improve the accuracy of ancestral state reconstructions with these two methods [47,53], neither parsimony nor ML can explicitly model directional trends, unlike analyses in Bayesian framework. Thus, we next addressed this issue and tested whether body size, brain size and encephalization evolved under directional trends in primates.

### Evolutionary trends in body and brain mass evolution

We tested for evolutionary trends by comparing a directional random-walk model to the non-directional random-walk model in BayesTraits. The implementation of the directional model requires variation in root-to-tip branch length [3,55,65] which in our otherwise ultrametric tree is provided by the inclusion of fossil data. To explicitly test for directionality in encephalization rather than simply inferring this from the evolutionary histories of brain and body mass, we used the residuals of brain size on body size of the species (computed as explained above with the *residuals first *approach) as species data of relative brain size in this analysis. The *residuals second *approach, in fact, could not be used in this context since it is based on residuals computed at internal nodes of the phylogeny.

**Figure 5:**
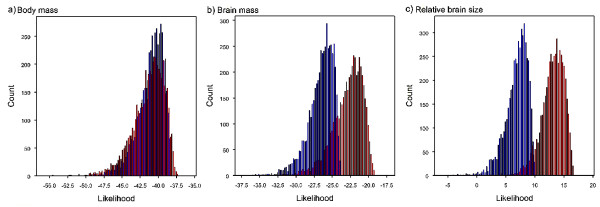
**Posterior distributions of log-likelihoods for the non-directional and directional models**. Figure **a) **shows body mass; **b) **brain mass; **c) **relative brain size. The log-likelihood of the directional model is shown in red, the non-directional model in blue. The posterior distributions of ancestral state estimates were obtained using uniform priors, two million iterations and a sampling interval of 100 (see Methods). The harmonic means and Bayes Factors of the posterior distributions are given in Table 1.

We found no evidence for a directional trend in absolute body mass, as the directional model did not provide a better fit to the data when compared to the non-directional model (Table 1; Figure 5). Therefore, in agreement with other authors [46,66], we conclude that there is no evidence that Cope's Rule [67,68], which states body size tends to increase through time, applies to primates. In contrast there is strong evidence for a trend of increasing absolute and relative brain size (Table 1; Figure 5) suggesting that the expansion of the primate brain has been of major evolutionary significance across the modern primate phylogeny and throughout primate evolution.

**Table 1:** Tests for evolutionary trends^1^.

Phenotype	Harmonic mean Log(Lh): Constant-variance model	Harmonic mean Log(Lh): Directional model	Bayes Factor
Absolute body size	-44.688	-45.275	-1.174
Absolute brain size	-30.282	-27.087	6.390
Relative brain size	1.647	8.576	13.857

^1 ^The model with the highest log-likelihood is the best-fitting model.

To assess how the presence of a directional trend affects the accuracy of estimates made with a non-directional model we performed correlations between the results obtained from the directional and non-directional models. Correlations between the estimates made for absolute brain mass under the directional and non-directional models suggest no nodes are estimated less accurately than others under the non-directional constant-variance model (*r*_*s *_= 0.995, *P *< 0.001). However, the directional model tends to give lower estimates for all nodes (Additional file 1, Figure S1 a). For relative brain mass the r_s _between estimates under directional vs. non-directional model is lower (r_s _= 0.943, *P *< 0.001) and, while nodes are generally estimated as having lower values under the directional model (Additional file 1, Figure S1 b), the ancestral state reconstructions for the deepest nodestend to differ more drastically.

Taken together our results suggest that the ancestral state reconstruction procedure implemented in Bayesian framework following Organ *et al*. [55] might be more reliable in comparison to parsimony and ML methods, as it first identifies the best predictive model based on known data, and then uses such model to infer unknown ancestral states. In addition it can explicitly model directionality, and therefore we could identify a directional trend to increase in both absolute and relative brain mass - but not body mass - in primates. For the purposes of this paper, we conclude that the most reliable estimates are thus obtained with Bayesian analyses, under a non-directional random walk model for body mass and directional random walk for absolute brain mass (Additional file 1, Table S3). For a discussion of the rate parameters used in the final analyses see the Supplementary information and Additional file 1, Table S2.

**Figure 6:**
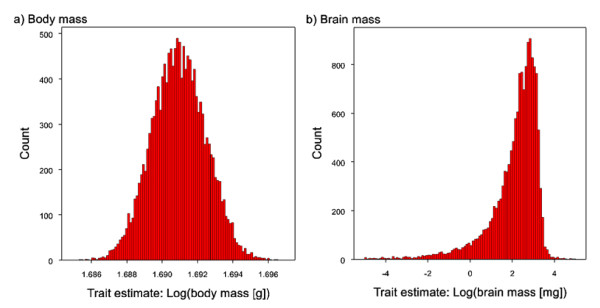
**Posterior distributions of trait estimates for the LCA of living primates for a) body mass and b) brain mass**. Histograms are plotted from a posterior distribution of ancestral state estimates obtained using uniform priors (prior range: -100 to +100) acceptance rates were within 20 to 40% (see methods). To ensure the chain fully explored the parameter space, we extended the MCMC run to 25 million iterations with a sampling interval of 1500.

Having obtained the most reliable estimates of ancestral states at each node in the tree for each phenotype it is then possible to use these to make evolutionary inferences. For example our most supported estimate of the body mass at the root of the primate tree using Bayesian analysis is largely consistent with some previous qualitative estimates. Martin [23] suggested the ancestral primate probably weighed less than 500 g, while Fleagle [45] used early primate fossils to conclude that the ancestral primate was probably as small as 20 g. This is similar to our estimated body mass at the root of the phylogeny obtained with the inclusion of fossil species (48.98 g, 95% CI: 48.97 g to 50.00 g; Figure 6). The estimate made without the fossils is similarly low (37.71 g, CI: 37.60 g to 37.76 g). Importantly, our estimate of body mass at the root lies within the range of the proposed extinct sister-group to modern primates, the plesiadapiforms, which ranged from 7 g (the *Micromomyidae *family) to 3,000 g (the *Carpolestidae *family), and is consistent with the estimated body masses of two putative early modern primates *Altanius *(10 g) and *Altiatlasius *(50 to 100 g) [45,69]. In contrast, a much higher estimate of ancestral primate body mass [1,171 g (95% CIs: 236 to 3,610 g)] was recently obtained using a parsimony method and extant species data only [46]. However, our results show that in the absence of fossil data parsimony leads to overestimates of body size, thus questioning this conclusion.

Body mass variation is associated with a number of behavioural, ecological and life history traits [44,45,70-75] which are frequently used to infer characteristics of extinct species. Small body mass in extant primates (less than 500 g) is usually associated with nocturnality, an insectivorous trophic niche [76] and leaping mode of locomotion for species weighing less than 3,000 g [45]. The probable small body mass of the ancestral primate has been interpreted as evidence that it occupied a fine-branch niche and was adapted for grasping small insect prey [23,77]. On the basis of correlates of body mass and ecological and life history traits of living primates [44,45], our estimated mass at the root would suggest the ancestral primate was a leaping insectivore, which might have had a lifespan of four to six years. This proposed ecology suggests that visual specialisation to meet the demands of a fine-branch, insect grasping niche may have had a significant role in the early expansion of the primate brain [18,23], a hypothesis consistent with recent evidence revealing an association between visual expansion and brain size in fossil endocasts of early primates [78].

Our reconstructions suggest the ancestral primate had a small brain (120.23 mg, 95% CI: 114.42 mg to 126.33 mg) which, in relative terms, was much smaller than in any living primate. This result is consistent with a study of a virtual endocast of *Ignacius graybullianus*, an Early Eocene Plesiadapiform (Paromomyidae), indicating that early euprimates exhibited only small advances in brain mass over ancestral groups and that the majority of increases in brain size occurred after the origin of modern primates [78]. Finally we note that our best estimate of brain mass for the last common ancestor of *Homo *and *Pan *(338.75 mg, 95% CI: 321.37 mg to 340.64 mg; which equates to a cranial capacity of 355.16 cm^3^, 95% CI: 336.61 cm^3 ^to 357.17 cm^3^) is similar to estimates for the two earliest hominids known from the fossil record, the 7.7 million year old *Sahelanthropus tchadensis *(360 to 370 cm^3^;) [79] and the 4.4 million year old *Ardipithecus ramidus *(280 to 350 cm^3^) [80]. These two fossil species were not included in our analysis due to uncertainty in their phylogenetic position which has only recently been resolved [80].

### Increases in brain mass in particular lineages

**Figure 7:**
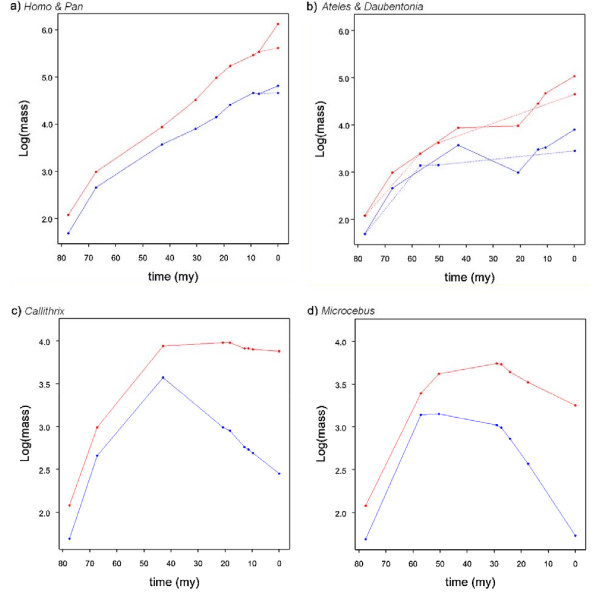
**Evolutionary trajectories of brain and body mass**. Evolution of brain (red) and body (blue) mass from the ancestral primate to **a) ***Homo *(solid line) and *Pan *(dashed line) and **b) ***Ateles *(solid line) and *Daubentonia *(dashed line) showing parallel increase in brain and body mass; **c) ***Callithrix*, and **d) ***Microcebus *demonstrating secondary reduction in both brain and body mass: note the reduction in brain mass is lower than the reduction in body mass leading to an increase in relative brain size (see Additional file 1, Table S4).

We next examined the amount of change along different branches of the tree, both as total change along the branches and as rate of change accounting for differences in branch lengths (see below). We first calculated the means of the posterior distribution of the ancestral states for each node, using the same posterior predictive model developed for brain and body mass, and we computed the change in absolute brain and body mass, and relative brain mass (using the *residuals second *approach described above) along each branch by comparing the values (either observed (Additional file 1, Table S1) or estimated (Additional file 1, Table S3)) at consecutive nodes. Our estimates show that both absolute and relative brain size have increased independently in all major clades of primates (examples shown in Figure 7a and 7b).

Changes in absolute brain mass along each branch of the phylogeny can be considered in two ways: a proportional increase (as % of increase relative to the ancestor) and an absolute increase in mass (described above). The average proportional change in absolute brain mass along a branch is 0.243 (i.e. a 24.3% increase), with changes greater than 0.344 being in the upper quartile, which includes branches from all the major clades of the phylogeny. Notably, three of the top four proportional increases are along the deepest branches (ancestral primate to ancestral strepsirrhine (node 38-node 65; see Figure 2), 1.310; ancestral primate to ancestral haplorhine (38 to 39), 0.942; ancestral haplorhine to ancestral anthropoid (39 to 40), 0.929), suggesting selective pressures favouring the expansion of the brain were strong early in primate evolution. Interestingly the proportional increase along the terminal human branch is only the seventh largest change (0.594). However in terms of absolute change the terminal human branch shows the largest change, almost four times greater than the second biggest change (the terminal *Pongo *branch).

The average proportional change in relative brain mass is 0.201 (that is, 20.1% increase), with changes above 0.278 falling in the upper quartile, which again includes branches from all major primate groups. The five branches which show the largest increase in relative brain mass are the terminal *Tarsius *branch (0.917), the terminal *Daubentonia *branch (0.837), the terminal *Galago *branch (0.514), the terminal human branch (0.479) and the branch between the last common ancestor of Catarrhines and PlatyrrhinesSimiformes and the ancestral Platyrrhine (40 to 54: 0.431). Because *Daubentonia *and *Tarsius *are no more gregarious than their close relatives [81], this suggests that social complexity is unlikely to have been the sole factor in primate brain mass evolution [c.f [7]] and that other selective pressures have also been important. For example the large brain of *Daubentonia *is partly due to olfactory specialisation [82,83] which is consistent with selection acting on sensory systems having had a significant role in brain size evolution [84]. The finding that the human branch only comes fourth by this measure is perhaps surprising, but we note that of these five branches the terminal human branch is the shortest.

We next examined evolutionary changes along branches controlling for branch length (change relative to time). The average rates were an increase of 0.025/million years (that is, a 2.5% increase/million years) for a proportional change in brain mass, 5,640 mg/million years for an absolute change in brain mass, and 0.020/million years for a change in relative brain mass confirming that most change in relative brain mass was due to brain rather than body mass. The branch with the highest rate of change in absolute brain mass is the terminal human branch (140,000 mg/million years). However for rate of proportional change in absolute brain mass the human branch comes only fourth, below the branches between the last common ancestor of Macaques and other *Papionini*, and the last common ancestor of baboons, mangabeys and mandrills (48 to 49), the ancestral primate and ancestral haplorhine (38 to 39) and the branch between the last common ancestor of *Cebinae, Aotinae *and *Callitrichidae*, and the ancestral *Cebinae *(58 to 60). The rate of change in relative brain mass along the human branch (0.068/million years) is also exceeded by the branch between the last common ancestor of *Alouatta*, *Ateles *and *Lagothrix *with the last common ancestor of *Ateles *and *Lagothrix *(branch 55 to 56; 0.73), the branch connecting the last common ancestor of *Cebinae, Aotinae *and *Callitrichidae*, and the ancestral *Cebinae *(branch 58 to 60; 0.074/million years) and the branch connecting the last common ancestor of the *Papionini *with the last common ancestor of *Papio*, *Mandrillus *and *Cercocebus *(branch 48 to 49; 0.084). We therefore conclude that only in terms of absolute mass and the rate of change in absolute mass has the increase in brain size been exceptional along the terminal branch leading to humans. Once scaling effects with body mass have been accounted for the rate of increase in relative brain mass remains high but is not exceptional.

It is also notable that the estimated brain size of the last common ancestor of modern primates is smaller relative to body size than any living species and that the expansion of the primate brain began early, with the deepest branches (for example, 38 to 39; 38 to 65; 39 to 40) ranking in the upper quartile in terms of both increases in absolute and relative brain mass (Additional file 1, Table S4).

### Decreases in brain mass and evolutionary scenarios for *H. floresiensis*

Despite both absolute and relative brain mass showing strong and significant evolutionary trends to increase, we find several branches go against this trend (examples shown in Figure 7c and 7d; Additional file 1, Table S4). Absolute brain mass decreases on approximately 14% of branches (10/70); independent decreases are observed in Old World monkeys (the terminal *Cercocebus *branch); in New World monkeys - several branches in the callitrichids, supporting the conclusion that this family has evolved by a process of phyletic dwarfism [85]; and in strepsirhines - several branches in the lemur clade. Branches on which there is an overall decrease in absolute brain mass account for approximately 6% of the total evolutionary time covered by the phylogeny used in this analysis. In all cases a decrease in absolute brain mass is accompanied by a decrease in absolute body mass. Body mass decreases much more frequently: 46% of branches show a decrease, accounting for 47% of the total evolutionary time covered. Decreases in relative brain mass occur less frequently, with only 4% (3/70) of branches showing a decrease in relative brain mass, representing only 2.1% of evolutionary time. Decreases in relative brain mass mostly appear to be linked to body mass increasing to a greater extent than brain mass. This, for example, provides some support for the hypothesis that small relative brain size in gorillas reflects increased *somatisation *rather than decreased encephalization [[86]; but see [87]].

To assess whether the proposed evolution of *Homo floresiensis *is consistent with observed decreases in brain mass which have occurred elsewhere in the primate phylogeny, we calculated the ratio change in brain and body mass [(brain descendant - brain ancestor)/(body descendant-body ancestor)] for branches showing a decrease, in order to facilitate comparisons with the literature (see methods). We used log values to take variation in body mass into account; decreases here are therefore proportional decreases in absolute mass. Our aim in this analysis is not to estimate the probability or likelihood of the evolution of a hominin with reduced brain and body mass but rather to test whether or not decreases seen during the evolution of *Homo floresiensis *fall within the range of other observed decreases in Primates. First we consider the evolution of the *H. floresiensis *brain assuming descent from a known hominin by insular dwarfism, a widely cited hypothesis [15,16,39,40]. For the 10 branches which showed a decrease the average ratio was 0.265 and the range was 0.006 to 0.825. We calculated the same ratio using *H. floresiensis *(estimated brain mass c. 380 g [26,31]) and three possible ancestral forms of *Homo erectus*, following Martin *et al*. [39]: *Homo erectus *broadly defined, Ngandong *H. erectus*, and Dmanisi hominins. We also include *Homo habilis *which has not been ruled out as a possible ancestor [36]. The change in brain size and the ratio of the change in brain and body mass were calculated for the two extreme values of body mass estimated for *H. floresiensis *(16 and 32 kg) [26] and their midpoint. In addition we used the brain/body mass scaling relationships [(brain descendant - brain ancestor)/(body descendant-body ancestor)] during the 10 decreases in brain mass to estimate the decrease in brain mass expected for the observed decrease in body mass for each ancestor and body mass. The results of this analysis are shown in Additional file 1, Table S5 and invoke similar conclusions to those discussed below.

Under a number of scenarios the evolution of *H. floresiensis *lies within the range of decreases in brain mass estimated here (Table 2). For any ancestor, except Ngandong hominoids, and a *H. floresiensis *body mass of 16 kg the decreases in brain and body mass always fall within the range of the decrease that we found in other primate branches. For a *H. floresiensis *body mass of 32 kg the decrease in relative brain mass is not consistent with changes estimated in other branches, but assuming a body mass estimate of 24 kg and descent from a Dmanisi hominin population, the decrease in relative brain size falls within the range of decreases observed elsewhere in the primate phylogeny. We also note that for both body mass estimates the proportional change in absolute brain mass from either a Dmanisi hominin (-0.216) or *H. habilis *(-0.137)ancestor is actually smaller than the decrease in the terminal *Microcebus *branch (-0.273). Finally the calculated change in relative brain mass from any of the four ancestors is compatible with the results obtained here only for a *H. floresiensis *body mass close to 16 kg, or descent from either a Dmanisi hominin or *H. habilis *if *H. floresiensis *had a body mass towards 24 kg.

**Table 2:** Evolution of brain size during the evolution of *H. floresiensis *from four possible ancestors by insular dwarfism.

Ancestor	*H. floresiensis *body mass (kg)	Ratio of change in log(absolute brain mass) & log(body mass)^2^	Change in log(brain mass)	Change in log(relative brain mass)
*H. erectus*	16	0.720*	-0.398	-0.020*
	24	1.058	-0.398	-0.141
	32	1.586	-0.398	-0.226
Ngandong	16	0.784	-0.450	-0.058
	24	1.131	-0.450	-0.178
	32	1.649	-0.450	-0.264
Dmanisi	16	0.437*	-0.216*	0.122*
	24	0.678*	-0.216*	0.002*
	32	1.116	-0.216*	-0.084
*H. habilis*	16	0.420*	-0.137*	0.059*
	24	0.908	-0.137*	-0.034
	32	5.219	-0.137*	-0.147

^1 ^computed as: [(brain descendant - brain ancestor)/(body descendant-body ancestor)]

* indicates a result which falls within the range of decreases in brain size for Primates estimated in this study.

Thus under the insular dwarfism model, if *H. floresiensis *descended from either an 'average' *H. erectus *or Ngandong populations, the decrease in brain mass is not compatible with our results unless *H. floresiensis *had a body mass near 16 kg. The evolution of *H. floresiensis *also appears less likely if it had a body mass towards the upper estimate, as the decrease in relative brain mass falls outside our estimates on other branches. However, if *H. floresiensis *had a body mass of 16 to 24 kg, descent from either a Dmanisi hominin or *H. habilis *ancestor is in line with decreases in brain and body mass along other primate lineages. We therefore conclude that further studies addressing the affinities of *H. floresiensis *with different possible ancestors and more accurate predictions of body mass are necessary to rule out the possibility of *H. floresiensis *being a true novel hominin using this kind of analysis. Our analysis suggests it is possible that, under the insular dwarfism model, the only unexpected aspect of *H. floresiensis' *evolution is the rate at which brain mass decreased, however some evidence suggests morphological evolution may accelerate on islands [88].

Next we performed several analyses to test whether the evolution of the *H. floresiensis *brain under the alternative phylogenetic scenario proposed by Argue *et al*. [43] by estimating the ancestral brain and body masses of the node at the base of the *H. floresiensis *lineage (Additional file 1, Table S6) and subsequently analysing the evolution of brain size along that lineage. The analysis was run for each of the two most parsimonious trees separately and then for both trees together, taking advantage of BayesTraits ability to take phylogenetic uncertainty into account. The results again suggest that if *H. floresiensis *body mass did not greatly exceed 24 kg the decrease in brain size observed along the lineage leading to *H. floresiensis *falls within the range seen elsewhere in the primate phylogeny, scales with body mass in a way consistent with other episodes of brain mass reductions, and actually results in an increase in relative brain size (Table 3; Additional file 1, Table S7). Conversely, a larger body size produces an allometric decrease in brain size beyond the range observed in the primate tree. To conclude, for a body mass toward the lower end of the range of estimates all the phylogenetic hypotheses on the ancestry of *H. floresiensis *so far proposed are consistent with the observed decrease in brain size. An alternative method of these hypotheses based on using the model of brain evolution developed in BayesTraits is presented in the supplementary information, and produces results broadly consistent with the main analysis.

**Table 3:** Evolution of brain size during the evolution of *H. floresiensis *under two phylogenetic scenarios^1^.

Ancestor	*H. floresiensis *body mass (kg)	Ratio of change in log(absolute brain mass) & log(body mass)^2^	Change in log(brain mass)	Change in log(relative brain mass)
Argue Tree 1	16	0.400*	-0.171*	0.121*
	24	0.639*	-0.171*	0.012*
	32	1.116	-0.171*	-0.066
Argue Tree 2	16	0.428*	-0.173*	0.104*
	24	0.709*	-0.173*	-0.006*
	32	1.336	-0.173*	-0.084
Both trees	16	0.418*	-0.176*	0.110*
	24	0.679*	-0.176*	-0.001*
	32	1.225	-0.176*	-0.076

^1 ^These are the two most parsimonious topologies obtained by Argue *et al*. [43] and are shown in Figure 1 b (tree 1) & c (tree 2)

^2 ^computed as: [(brain descendant - brain ancestor)/(body descendant-body ancestor)]

* indicates a result which falls within the range of decreases in brain size for Primates estimated in this study.

To further study the selective pressures and anatomical changes associated with decreases in brain mass we suggest *Microcebus*, *Callithrix *and *Miopithecus *or *Cercocebus *may be useful, independent models. For example, Falk *et al*. [31] identified a number of potentially derived features in an endocast of *H. floresiensis*, and comparative analyses of the brain anatomies of these species might show whether similar structures are modified in independent episodes of brain mass reduction. Likewise a comparative analysis of the ecologies of these smaller brained primates may reveal selective pressures associated with decreases in brain and body mass. We note that for both decreases in absolute and relative brain mass there appears to be no relation with isolation on islands, nor is there any clear single ecological trait that can explain these decreases. As with evolutionary increases in brain mass, decreases in mass are likely to be influenced by a number of ecological factors. For example, Taylor & van Schaik [24] have shown that brain size has decreased during the evolution of *Pongo p. morio*, particularly in females. These authors suggest this reduction is associated with an increase in periods of food scarcity resulting in selection to minimise brain tissue which is metabolically expensive [17]. Food scarcity is also believed to have played a role in the decrease in brain size in the island bovid *Myotragus *[12]. Taylor & van Schaik [24] therefore propose that *H. floresiensis *may have experienced similar selective pressures as *Myotragus *and *Pongo p. morio*. Future studies are needed to address the relative contributions of proposed social and ecological factors in both decreases and increases in brain mass across Primates and other species.

## Conclusions

By reconstructing ancestral states of brain and body mass in primates we have shown that Organ's et al [55] method, implemented in BayesTraits using Bayesian analysis, is least affected by the inclusion of fossil data and is therefore more reliable for our dataset. In this respect this approach outperforms parsimony and ML methods which instead tend to produce lower estimates at deep nodes when using only data of extant species. This is likely to be because BayesTraits first identifies the best predictive model based on known tip data to then infer unknown ancestral states at each node of interest in the tree and can incorporate evolutionary trends. If Organ *et al*.'s [55] method generally outperforms ML and parsimony methods, this may have important implications for future studies which attempt to estimate ancestral states in groups where little fossil information is available, or where evolutionary trends are suspected, especially when the reconstruction is performed on deep nodes within phylogenies which cover large time periods. Studies on datasets with known ancestral states are thus needed to fully assess if the method implemented in BayesTraits consistently produces more reliable ancestral state reconstructions.

Our results provide robust confirmation for the suggestion that strong evolutionary trends have governed the expansion of the primate brain. In contrast body size evolution has not tended to increase in primates, implying brain and body mass have been subject to separate selection pressures and supporting the findings of previous studies in other taxonomic groups that these two highly correlated traits can show differences in their patterns of evolution [89,90]. In primates, brain mass has independently expanded in both absolute and relative terms in all the major clades of the primate phylogeny and began to increase early in primate evolution. We have highlighted branches along which the change or rate of change in brain mass is particularly large. Surprisingly only in terms of change in absolute mass is the terminal human branch exceptional; once scaling effects are accounted for, humans rank only seventh. Despite the presence of an overall trend to increase mass, we also provide evidence for independent decreases in brain mass in New and Old World Monkeys and in strepsirhines. From our analyses of evolution of *H. floresiensis *brain size under different phylogenetic hypotheses, we conclude that the evolution of *H. floresiensis *is consistent with our results across the primate phylogeny if it either evolved from populations of *H. habilis *or Dmanisi hominin by insular dwarfism, or under Argue *et al*.'s [43] proposed phylogenetic scenarios, and if *H. floresiensis *had a body mass towards the lower end of the range of estimates obtained from skeletal remains. In this respect we note that Brown *et al*. [26] suggested the lower body mass estimates are probably most appropriate, assuming *H. floresiensis *shared the lean body shape typical of Old World tropical modern humans. If this were true we estimate the evolution of *H. floresiensis *involved a reasonable decrease in absolute brain mass, but an increase in relative brain size. Our analysis, together with studies of brain size in island populations of living primates[41,42], therefore suggests we should perhaps not be surprised by the evolution of a small brained, small bodied hominin, although further clarification of the relationships between *H. floresiensis *and other hominins are required to confirm this observation. Finally, our analyses add to the growing number of studies that conclude that the evolution of the human brain size has not been anomalous when compared to general primate brain evolution [59,61,91-94].

## Methods

### Brain and body mass data

Data for body and brain mass were obtained from previously published datasets [22,82,95]. For reconstructions of ancestral brain and body mass, we used data from as many extant genera as possible, leading to a dataset of 37 primate genera including 14 catarrhines, 12 platyrrhines, 1 tarsier and 10 strepsirhines (Additional file 1, Table S1a).

Through a literature search we obtained data for fossils where cranial remains were sufficiently intact to make reliable estimates of cranial capacity (N = 23, Additional file 1, Table S1b). We converted cranial capacity to brain mass using the equation given in Martin [23]: *Log(cranial capacity) = [1.018 × Log(brain mass)] - 0.025*. Where body and brain mass estimates were not available from the same individual we took the body mass estimate for the species given in Fleagle [45]. The dataset includes seven extinct hominins, which we use to examine whether ancestral values were overly influenced by the large disparity between the brain mass of *Homo sapiens *and the other apes.

To calculate relative brain mass we performed a phylogenetically-controlled regression analysis (see below) between log(brain mass) and log(body mass) in BayesTraits [54,96]. In all the analyses using Generalised Least Squares models (GLS), the phylogeny is converted into a variance-covariance matrix representing the shared evolutionary path between the species [3,54,65]. The GLS regression analysis was performed with ML and MCMC. Bayesian MCMC analyses were completed using uniform priors (prior range -100.00 to 100.00), with 2,000,000 iterations and a sampling period of 100, after a burn in of 500,000. The rate deviation was set to obtain an average parameter acceptance rate of 20 to 40%. We first identified, in Bayesian framework [97,98], the regression model that best described the relationship between brain and body mass, by testing whether additional branch-length scaling parameters to the default Brownian motion model improved the fit to the data. These were lambda, which reveals to what extent the phylogeny predicts the pattern of covariance between species for a trait (the phylogenetic signal); kappa, which stretches and compresses branch lengths; and delta, which scales path lengths. As these parameters can improve the fit to the data, we first estimated all parameters at once following Organ *et al*. [55] and, where a parameter was significantly different from the default value of 1, indicating gradualism, it was then estimated in the final regression analysis. However, while lambda can be estimated alone, kappa and delta are better estimated as additional parameters in the model that also included lambda. The regression was highly significant (t_59 _= 14.53, R^2 ^= 0.858, *P *< 0.001), the branch-length scaling parameters lambda and delta were not significantly different from the default value of one (lambda: lambda = 0.979, LR = 2.04, *P *= 0.153; delta: delta = 1.091, LR = 0.03, *P *= 0.857). Conversely, kappa was estimated to be 0.474, significantly different to one (LR = 8.132, *P *= 0.004).

Relative brain mass on body mass for each species (extant or extinct) was calculated as residual values using the regression equation (see below). These residuals were used to test for an evolutionary trend to increase relative brain mass and to reconstruct ancestral states (*residuals first*).

### Phylogeny

It is important to incorporate both topology and branch length information during reconstruction analyses as species are part of a hierarchically structured phylogeny, therefore not statistically independent, and differences in time since divergence from the common ancestors determines differential potential for evolutionary change [3,47,99,100]. We used a genus level composite phylogeny of primates using published trees. The topology is taken from Goodman *et al*. [101] for haplorhine primates and Horvath *et al*. [102] for strepsirhines. Proportional branch lengths were obtained from recent studies of primate divergence dates [103-106] scaled to agree with dates of divergence for the deeper primate nodes estimated by Steiper & Young [107]. The tree obtained therefore has branch length information and is ultra-metric. There are two trichotomies: one between the *Cebidae*, *Pitheciidae *and *Atelidae*, the other at the base of *Cebidae *(Figure 2). As the topology of our composite phylogeny is well studied and the branch lengths are based on the best available divergence date estimates in all subsequent analyses it is assumed our phylogeny is known without error.

Where fossil data were included we follow Finarelli & Flynn [47] in minimising phylogenetic assumptions and placed extinct taxa as polytomies at the node nearest to their estimated position in the primate phylogeny. Branch lengths for fossil species were calculated as the time from this node to the end of the geological period in which they are last found. Both phylogenetic relationships and temporal presence in the fossil record were taken from Fleagle [45]. Where the programs described below require a fully bifurcating tree, trichotomies were randomly resolved and the new, intervening branch given a branch length of zero.

We also use the two most parsimonious *Hominin *topologies obtained by Argue *et al*. [43]. Here branch lengths were determined based on the earliest and latest known fossils for each species [27,45,108,109], with divergence dates of internal nodes coming from the first appearance of any species within the lineages which evolved from that node. Where the time of origin for a lineage could not be determined in this way we minimise phylogenetic assumptions by placing in the node in centre of the branch. The rest of the phylogeny was identical to that presented in Figure 1[110].

### Reconstruction methods

Ancestral state reconstructions of absolute brain and body mass and relative brain mass at each node of the phylogeny were estimated using three methods: weighted squared-change parsimony in the Mesquite [101], ML in the program ANCML [63], and Bayesian MCMC [97,98], in BayesTraits following Organ *et al*.'s [55] method, all of which incorporate phylogenetic information

Weighted squared-change parsimony infers ancestral states by minimising the square-change along branches [62,63], but parsimony approaches are not robust to violations of assumptions of constant rate of evolution or equal probability of change in either direction [3,51,64,111]. Throughout the paper we refer to this method as Parsimony method.

ML reconstruction is based on a Brownian motion model to estimate transitions at any node along the phylogeny. The advantages of this method are that the probability of change at any point in the tree is not dependent on a prior state change or on changes on other branches [2]. Like parsimony approaches however, the model assumes a constant rate of evolution and may perform poorly if the trait shows an evolutionary trend [2,51,64] (see below). Throughout the paper we refer to this method as the ML method.

Finally, the ancestral state reconstructions of brain and body size were performed in Bayesian framework with MCMC in BayesTraits [3,54], following the method described in Organ *et al*. [55]. Throughout the paper we refer to the results obtained from BayesTraits as the Bayesian analysis. BayesTraits first identifies the best fitting evolutionary model (see below) to the species data, and then uses such model to infer unknown ancestral states at internal nodes along the tree. The ancestral state reconstruction is therefore performed in two steps. The constant variance random walk model has only one parameter, alpha, which describes the instantaneous variance of evolution [3,65]; this model represents the default model with all branch length scaling parameters, kappa, lambda and delta, set as 1 [3,65]. We then simultaneously estimated the branch length scaling parameters, because these parameters can improve the fit to the data and thus help identify the best evolutionary model for the data. We tested whether the branch scaling parameters differed from the default value of one by comparing the harmonic mean of the model in which the parameters were estimated to the harmonic mean of the model where they were set as one, using Bayes factors. The Bayes Factor is computed as: *2(log [harmonic mean(directional model) - log[harmonic mean(constant variance model)]*. A positive Bayes factor greater than two is taken as positive evidence for a difference between the two models with the best fitting model having the highest log(harmonic mean), a Bayes factor greater than five represent *strong *evidence and greater than 10 is *very strong *evidence [97]. Thus, the default value of one for a scaling parameter was used in the final analysis when the Bayes factor was less than two. Where they did, the rate parameters were incorporated in the estimation for the best fitting model (see supplementary information). Therefore, contrary to parsimony and ML, this method has the advantage of finding the best model of trait evolution before estimating ancestral states.

Results of the Bayesian analysis obtained using 2 or 10 million runs were qualitatively similar, therefore we performed all analyses with two million runs. All analyses were performed using uniform priors (prior range: -100 to +100), with 2,000,000 MCMC runs after a burn-in of 500,000, sampling every 100 runs, and repeated multiple times to test the stability of the harmonic means. Rate deviation was adjusted to obtain an acceptance of the proposed model parameters (above) between 20% and 40%. Ancestral state reconstructions were then simultaneously estimated using the best evolutionary model for the data; data deviation was adjusted to obtain an acceptance rate for each node's estimate between 20 to 40%.

Next we tested if a directional-change random walk model improved the fit to the data relative to the best non-directional random walk (Brownian motion) model obtained as described above. While the non-directional random walk model has one parameter - alpha, the variance of evolution - the directional random-walk model has an additional parameter that captures the directional change using a regression between trait values and the total path length (beta) [3,65]. Because all species have the same total path length in ultrametric trees, this analysis could be done only with the tree that incorporated fossil species as the directional model requires root-to-tip (path length) variation in order to estimate directionality unless the scaling parameters are used. The harmonic mean of the likelihoods of the directional and non-directional random walk models can be compared with Bayes factors [97,98] to determine which model fits the data best (see above).

ANCML (ML analysis) provide standard errors for each nodal value reconstruction. However, some authors consider standard errors to be underestimated and difficult to compare across methods [52]. The vast majority of posterior distributions of estimates made by the Bayesian MCMC runs were normally distributed using a W-test, so we present the mean and 95% confidence intervals for each node. To test for the sensitivity of each method to the inclusion of fossil species, we followed Webster & Purvis [64] and checked the strength of the association between estimates at each node made with and without fossil data using correlation analysis in GenStat (VSN^i^, Hemel Hempstead, UK). For the Bayesian analysis we thus calculated the mean of the posterior distribution of the ancestral states at each node of the tree. Because some sets of estimates made with different methods were not normally distributed, we used Spearman's rank correlation for all tests to allow correlation coefficients (*r*_*s*_) to be fully comparable throughout the analysis.

The change in brain and body mass along each branch was calculated by taking the difference between consecutive nodes. As the estimates for each node using absolute values of log brain and body mass are log values, subtracting consecutive node values gives a proportional change in mass. We therefore also converted log values into absolute numbers before calculating differences to get the absolute change in mass. Estimates of ancestral relative brain mass are based on residual values from a regression analysis of two log values. We therefore simply subtracted successive nodes to calculate change in relative brain mass. Finally, to control for differential potential in divergence due to longer time since the last splitting event we repeated the analysis and calculated the rate of change by dividing the change along a branch by the branch length, for each measure of brain mass.

## Abbreviations

CI: Confidence Interval; GLS: Generalised Least Squares; MCMC: Markov-Chain Monte Carlo; ML: Maximum Likelihood.

## Authors' contributions

SHM, NIM, IC and RAB designed the study, SHM performed the study, SHM, NIM, IC and RAB wrote the manuscript.

## Supplementary Material

Additional file 1 **Supplementary tables and figures**. 1. Table S1: Brain and body mass of primates used in the analyses. 2. Table S2: Posterior distribution of the scaling parameters to identify the best model before reconstructing ancestral states in Bayesian analysis. 3. Figure S1: Correlations between estimates made using directional constant variance random walk and non-directional constant variance random walk models in BayesTraits. 4. Table S3: Ancestral state estimates using most supported models. 5. Table S4: Change in absolute brain and body mass and relative brain mass along each branch. 6. Additional analyses in relation to *H. floresiensis*: • Table S5: Range of estimated decreases in brain mass during the evolution of *H. floresiensis *given scaling relationships during episodes of brain mass reduction. • Table S6: Estimated Log(body) and Log(brain) masses for the node at the base of the *H. floresiensis *terminal branch using the topologies proposed by Argue *et al*. [43]. • Table S7: Range of estimated decreases in brain mass during the evolution of *H. floresiensis *using the topologies proposed by Argue *et al*. [43] and given scaling relationships during brain mass reduction in primates. Click here for file
