# Supplementary material for: Reconstructing the ups and downs of primate brain evolution: implications for adaptive hypotheses and Homo floresiensis
Source: BMC Biol. 2010 Jan 27;8:9. doi: 10.1186/1741-7007-8-9 (PMC2825212; doi:10.1186/1741-7007-8-9)
Supplement: Additional file 1 — Supplementary tables and figures. 1. Table S1: Brain and body mass of primates used in the analyses. 2. Table S2: Posterior distribution of the scaling parameters to identify the best model before reconstructing ancestral states in Bayesian analysis. 3. Figure S1: Correlations between estimates made using directional constant variance random walk and non-directional constant variance random walk models in BayesTraits. 4. Table S3: Ancestral state estimates using most supported models. 5. Table S4: Change in absolute brain and body mass and relative brain mass along each branch. 6. Additional analyses in relation to H. floresiensis: • Table S5: Range of estimated decreases in brain mass during the evolution of H. floresiensis given scaling relationships during episodes of brain mass reduction. • Table S6: Estimated Log(body) and Log(brain) masses for the node at the base of the H. floresiensis terminal branch using the topologies proposed by Argue et al. [43]. • Table S7: Range of estimated decreases in brain mass during the evolution of H. floresiensis using the topologies proposed by Argue et al. [43] and given scaling relationships during brain mass reduction in primates. [file 1741-7007-8-9-S1.DOC]

# Reconstructing the ups and downs of primate brain evolution: implications for adaptive hypotheses and *Homo floresiensis*

### Stephen H Montgomery, Isabella Capellini, Robert A Barton, Nicholas I Mundy

**Supplementary tables & figures.**

**Contents:**

**1. Table S1 -** Brain and body mass of primates used in the analyses.

**2. Table S2:** Posterior distribution of the scaling parameters to identify the best model before reconstructing ancestral states in Bayesian analysis.

**3. Figure S1:** Correlations between estimates made using directional constant variance random walk and non-directional constant variance random walk models in BayesTraits.

**4. Table S3:** Ancestral state estimates using most supported models.

**5. Table S4:** Change in absolute brain and body mass and relative brain mass along each branch.

**6. Additional analyses in relation to *H. floresiensis***

- **Table S5:** Range of estimated decreases in brain mass during the evolution of *H. floresiensis* given scaling relationships during episodes of brain mass reduction.
- **Table S6:** Estimated Log(body) and Log(brain) masses for the node at the base of the *H. floresiensis* terminal branch using the topologies proposed by Argue *et* al. [55].
- **Table S7:** Range of estimated decreases in brain mass during the evolution of *H. floresiensis* using the topologies proposed by Argue *et* al. [55] and given scaling relationships during brain mass reduction in primates.
- **Table S8:** Predicted Log(brain mass) of *H. floresiensis* under a number of phylogenetic scenarios.

**1. Table S1 - Brain and body mass of primates used in the analyses.**

| **A) Extant Primates** | **Brain size (mg)** | **Body size (g)** | **Relative brain mass^1^** | **Reference(s)** |
| --- | --- | --- | --- | --- |
| *Homo sapiens* | 1330000 | 65000 | 0.653 | 82 |
| *Pan troglodytes* | 405000 | 46000 | 0.239 | " |
| *Gorilla gorilla* | 500000 | 105000 | 0.086 | " |
| *Pongo pygmaeus* | 413300 | 57021.5 | 0.184 | 95 |
| *Hylobates lar* | 102000 | 5700 | 0.261 | 82 |
| *Papio anubis* | 201000 | 25000 | 0.116 | " |
| *Mandrillus sphinx* | 179000 | 32000 | -0.007 | " |
| *Cercocebus aligena* | 104000 | 7900 | 0.172 | " |
| *Macaca mulatta* | 93000 | 7800 | 0.127 | " |
| *Cercopithecus aetiops* | 73200 | 4819 | 0.166 | " |
| *Erythrocebus patas* | 108000 | 7800 | 0.192 | " |
| *Colobus badius* | 78000 | 7000 | 0.083 | " |
| *Presbytis entellus* | 119400 | 21319 | -0.063 | " |
| *Pygathrix nemaeus* | 77000 | 7500 | 0.057 | " |
| *Alouatta sp.* | 52000 | 6400 | -0.066 | " |
| *Lagothrix lagotricha* | 101000 | 5200 | 0.284 | " |
| *Ateles geoffroyi* | 108000 | 8000 | 0.185 | " |
| *Cebus sp.* | 71000 | 3100 | 0.284 | " |
| *Saimiri sciureus* | 24000 | 660 | 0.272 | " |
| *Aotus sp.* | 17100 | 830 | 0.057 | " |
| *Saguinus oedipus* | 10000 | 380 | 0.056 | " |
| *Leontopithecus rosalia* | 13400 | 590 | 0.053 | 22 |
| *Callimico goeldii* | 11000 | 480 | 0.028 | 85 |
| *Callithrix jacchus* | 7600 | 280 | 0.028 | " |
| *Callicebus moloch* | 19000 | 900 | 0.079 | " |
| *Pithecia monacha* | 35000 | 1500 | 0.192 | " |
| *Tarsius sp.* | 3600 | 125 | -0.057 | " |
| *Galago senegalensis* | 4800 | 186 | -0.051 | " |
| *Loris tardigradus* | 6600 | 322 | -0.075 | " |
| *Nycticebus coucang* | 12500 | 800 | -0.068 | " |
| *Daubentonia madagascariensis.* | 45150 | 2800 | 0.118 | " |
| *Propithecus verreauxi* | 26700 | 3480 | -0.175 | " |
| *Lepilemur ruficaudatus* | 7600 | 915 | -0.324 | " |
| *Cheirogaleus major* | 6800 | 450 | -0.162 | " |
| *Microcebus murinus* | 1780 | 54 | -0.114 | " |
| *Varecia variegatus* | 31500 | 3000 | -0.059 | " |
| *Eulemur fulvus* | 23300 | 1400 | 0.036 | " |
|  |  |  |  |  |
| **B) Extinct primates** | **Brain size (mg)^2^** | **Body size (g)** | **Log-Log Residual** | **Reference(s)** |
| *Homo heidelbergensis* | 1118362 | 62000 | 0.592 | 108 |
| *Homo erectus* | 951228 | 57000 | 0.547 | 108 |
| *Homo ergaster* | 802015 | 58000 | 0.467 | 108 |
| *Dmanisi homoids* | 626362 | 50000 | 0.404 | 109 |
| *Homo habilis* | 522414 | 34000 | 0.440 | 108 |
| *Homo rudolfensis* | 707814 | 45000 | 0.488 | 108 |
| *Homo floresiensis* | 380723 | 16000-32000? | 0.320-0.526? | 27; 31 |
| *Paranthropus boisei* | 486134 | 44000 | 0.332 | 108 |
| *Australopithecus africanus* | 433953 | 36000 | 0.342 | 108 |
| *Proconsul africanus* | 161426 | 10500 | 0.279 | 23 |
| *Oreopithecus bambolii* | 383060 | 30000 | 0.342 | 112; 45 |
| *Victoriapithecus macinnesi* | 53250 | 4500 | 0.048 | 113 |
| *Anapithecus hernyati* | 107116 | 13500 | 0.026 | 114 |
| *Catopithecus browni* | 3215 | 900 | -0.693 | 115; 45 |
| *Parapithecus grangeri* | 11555 | 1800 | -0.343 | 116 |
| *Aegyptopithecus zeuxis* | 34194 | 67100 | -0.946 | 23 |
| *Chilecebus carrasoensis* | 7618 | 582.6 | -0.189 | 117 |
| *Tetonius homunculus* | 1576 | 74 | -0.261 | 23 |
| *Necrolemur antiquus* | 3927 | 233 | -0.205 | 23 |
| *Rooneyia viejaensis* | 7558 | 782 | -0.280 | 23 |
| *Mioeuoticus sp.* | 7959 | 1280 | -0.404 | 23 |
| *Notharctus tenebrosus* | 10559 | 1990 | -0.412 | 23 |
| *Smilodectes gracilis* | 9660 | 1960 | -0.446 | 23 |
| *Pronycticebus gaudryi* | 4940 | 1220 | -0.597 | 23 |
| *Adapis parisiensis* | 8961 | 2350 | -0.533 | 23 |

^1^ Relative brain size was calculated following the ‘residuals first approach’, as the residual from a phylogenetically controlled GLS regression analysis between log(brain mass) and log(body mass) for the extant species. The GLS regression was performed using ML in BayesTraits (see Methods), and returned the following fit line: log(brain mass) = 2.18+0.684[log(body mass)].

^2^ For fossil data where brain size is estimated as volume we present the data after conversion to mg using the equation given in Martin [23]: *Log(cranial capacity) =[1.018 x Log(brain mass)] – 0.025*

1. Strauss WL, Schön MA: **Cranial capacity of *Oreopithecus bambolii***. *Science* 1960, **132**:670-672.
2. Benefit BR, McCrossin: **Earliest known Old World Monkey Skull.** *Nature* 1997, **388**:368-371.
3. Nargolwalla MC, Begun DR, Dean MC, Reid DJ, Kordos L: **Dental development and life history in *Anapithecus hernyaki***. *J Hum Evol* 2005, **49**:99-121.
4. Simons EL, Rasmussen DT: **Skull of *Catopithecus browni*.** *Am J phys Anthrop* 1996, **100**:261–292.
5. Simons EL: **The cranium of *Parapithecus grangeri*, an Eygptian Oligocene anthropoidean primate**. *Proc Natl Acad Sci USA* 2001, **98**:7892-7897.
6. Sears KE, Finarelli JA, Flynn JJ, Wyss AR: **Estimating body mass in New World “monkeys” (Platyrrhini, Primates), with a consideration of the Miocene platyrrhine, *Chilecebus carrascoensis***. *American Museum Novitates* 2008, **3617**:1-29.

**2. Table S2:** **Posterior distribution of the scaling parameters to identify the best evolutionary model before reconstructing ancestral states in Bayesian analysis.**

Table S1 below shows the Maximum Likelihood and mean estimates with 95% confidence intervals from the Bayesian MCMC analysis using the constant-variance random walk model to identify the best model before the ancestral state reconstruction.

Table S2: estimation of rate parameters*

| **Trait** | **Analysis** | **Kappa** | **Delta** | **Lambda** |
| --- | --- | --- | --- | --- |
| Body mass | ML | 0.875 | 0.533 | 1.00 |
|  | Bayesian MCMC | 0.936 (±0.009) | 0.636 (±0.009) | 0.980 (±0.002) |
| Brain mass | ML | 0.533 | 0.528 | 1.000 |
|  | Bayesian MCMC | 0.704 (±0.013) | 0.740 (±0.017) | 0.978 (±0.004) |
| Relative brain size | ML | 0.456 | 0.381 | 0.998 |
|  | Bayesian MCMC | 0.660 (±0.012) | 0.497 (±0.016) | 0.915 (±0.009) |

* Results of Bayesian analysis shown as the mean with 95% confidence intervals

For none of the three traits was the ML estimates or Bayesian MCMC posterior distributions of lambda significantly different from the default value of one. We subsequently tested whether the posterior distributions of kappa and delta differed from the default value of 1 by comparing the harmonic mean of the model in which the parameter was estimated to the harmonic mean of the model where it was set as 1. The default value of 1 was used in the final analysis when the Bayes Factor was less than 2 [97]. For absolute body size neither kappa (Bayes Factor = 0.740) nor delta (Bayes Factor = 0.063) differed from 1. For absolute brain size both kappa (Bayes Factor = 3.00) and delta (Bayes Factor = 2.00) differed from 1. Finally for relative brain size both kappa (Bayes Factor = 5.753) and Delta (Bayes Factor = 6.192) differed from 1. The posterior distribution of kappa and delta were therefore estimated in the best-fitting model for absolute and relative brain size used to compare the non-directional and directional models and in the reconstruction analysis. As kappa and delta are better estimated with lambda we also estimated lambda in the final analysis of absolute and relative brain size.

**3. Figure S1: Correlations between estimates made using directional constant variance random walk and non-directional constant variance random walk models in BayesTraits.**

**a) Absolute brain mass** and **b) relative brain mass.**


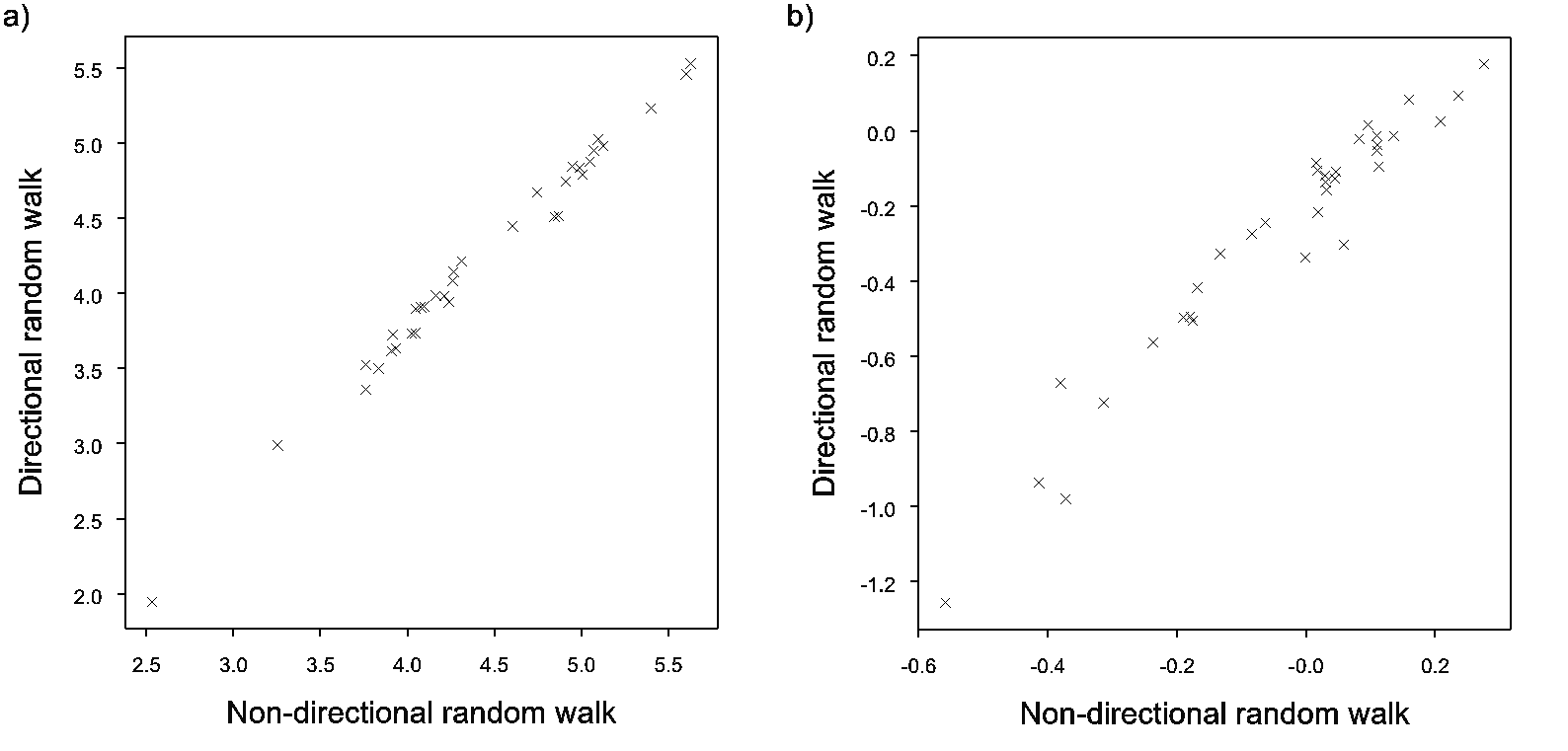


**4. Table S3: Ancestral state estimates using most supported models.^1^**

| **Node**  **(see Figure 2)** | **Log[Body mass (g)]** | | **Log[Brain mass (mg)]** | | **Relative brain mass** |
| --- | --- | --- | --- | --- | --- |
| 38 | 1.69 | (±0.0001) | 2.08 | (±0.0215) | -1.26 |
| 39 | 2.66 | (±0.0021) | 3.02 | (±0.0047) | -0.97 |
| 40 | 3.57 | (±0.0016) | 3.94 | (±0.0025) | -0.67 |
| 41 | 3.90 | (±0.0023) | 4.51 | (±0.0032) | -0.34 |
| 42 | 4.15 | (±0.0017) | 4.98 | (±0.0024) | -0.04 |
| 43 | 4.41 | (±0.0022) | 5.23 | (±0.0028) | 0.03 |
| 44 | 4.66 | (±0.0018) | 5.46 | (±0.0025) | 0.09 |
| 45 | 4.64 | (±0.0016) | 5.53 | (±0.0023) | 0.17 |
| 46 | 3.85 | (±0.0017) | 4.51 | (±0.0031) | -0.30 |
| 47 | 3.97 | (±0.0015) | 4.79 | (±0.0024) | -0.10 |
| 48 | 4.04 | (±0.0015) | 4.88 | (±0.0023) | -0.05 |
| 49 | 4.07 | (±0.0015) | 4.95 | (±0.0023) | -0.01 |
| 50 | 4.13 | (±0.0015) | 5.02 | (±0.0022) | 0.01 |
| 51 | 3.91 | (±0.0016) | 4.84 | (±0.0024) | -0.02 |
| 52 | 3.93 | (±0.0019) | 4.74 | (±0.0026) | -0.12 |
| 53 | 4.03 | (±0.0017) | 4.85 | (±0.0024) | -0.09 |
| 54 | 2.99 | (±0.0013) | 3.98 | (±0.0022) | -0.24 |
| 55 | 3.48 | (±0.0020) | 4.45 | (±0.0026) | -0.11 |
| 56 | 3.52 | (±0.0020) | 4.67 | (±0.0026) | 0.09 |
| 58 | 2.95 | (±0.0016) | 3.98 | (±0.0023) | -0.21 |
| 60 | 3.00 | (±0.0020) | 4.21 | (±0.0027) | -0.02 |
| 61 | 2.76 | (±0.0018) | 3.91 | (±0.0026) | -0.15 |
| 62 | 2.73 | (±0.0018) | 3.91 | (±0.0025) | -0.14 |
| 63 | 2.69 | (±0.0018) | 3.90 | (±0.0024) | -0.12 |
| 64 | 3.02 | (±0.0022) | 4.14 | (±0.0027) | -0.10 |
| 65 | 3.14 | (±0.0018) | 3.39 | (±0.0036) | -0.94 |
| 66 | 2.77 | (±0.0029) | 3.50 | (±0.0034) | -0.56 |
| 67 | 2.73 | (±0.0028) | 3.72 | (±0.0031) | -0.32 |
| 68 | 3.15 | (±0.0026) | 3.62 | (±0.0044) | -0.72 |
| 69 | 3.02 | (±0.0023) | 3.74 | (±0.0036) | -0.51 |
| 70 | 2.99 | (±0.0023) | 3.73 | (±0.0034) | -0.49 |
| 71 | 2.86 | (±0.0023) | 3.64 | (±0.0032) | -0.50 |
| 72 | 2.57 | (±0.0023) | 3.52 | (±0.0030) | -0.41 |
| 73 | 3.18 | (±0.0025) | 4.08 | (±0.0033) | -0.27 |

**^1^** Body mass ancestral values were estimated using a constant-variance random walk model, brain mass and relative brain mass by a directional constant-variance random walk model, following Organ et al.’s [55] method using MCMC analysis in BayesTraits, using the tree of extant and extinct species in Figure 1a. Estimates of body mass and absolute brain mass are given as the mean with 95% confidence intervals of the posterior distribution. The estimates of relative brain size are calculated as residuals of brain mass on body mass using the means of the posterior distribution of the reconstructed ancestral states of Log(brain) and Log(body) masses with the most supported models (previous two columns), and the phylogenetically controlled GLS regression equation (the ‘residuals second’ method: see main paper). Nodes refer to Figure 2.

**5. Table S4: Change in absolute brain and body mass and relative brain mass along each branch^1^**.

| **BRANCH** | **Change in absolute brain mass** | | | | **Change in body mass** | | | | **Change in relative brain mass** | |
| --- | --- | --- | --- | --- | --- | --- | --- | --- | --- | --- |
|  | **Proportional change** | **Rate**  **(/million years)** | **Absolute change (mg)** | **Rate**  **(mg/million years)** | **Proportional change** | **Rate**  **(/million years)** | **Absolute**  **change (g)** | **Rate**  **(g/million years)** | **Change** | **Rate**  **(/million years)** |
| **40..41** | 0.565 (±0.0033) | 0.039 | 23400 | 1610 | 0.334 (±0.0026) | 0.023 | 4270 | 294 | 0.330 | 0.023 |
| **41..42** | 0.473 (±0.0032) | 0.061 | 63300 | 8220 | 0.247 (±0.0024) | 0.032 | 6080 | 790 | 0.305 | 0.040 |
| **42..43** | 0.253 (±0.0029) | 0.050 | 75700 | 14800 | 0.259 (±0.0021) | 0.051 | 11400 | 2240 | 0.067 | 0.013 |
| **43..44** | 0.222 (±0.0031) | 0.026 | 114000 | 13400 | 0.257 (±0.0024) | 0.030 | 20600 | 2410 | 0.061 | 0.007 |
| **44..45** | 0.074 (±0.0027) | 0.036 | 53300 | 25739 | -0.022 (±0.0015) | -0.011 | -2300 | -1110 | 0.084 | 0.040 |
| **45..*Homo*** | 0.594 (±0.0023) | 0.084 | 991000 | 140000 | 0.172 (±0.0016) | 0.024 | 21200 | 3000 | 0.479 | 0.068 |
| **45..*Pan*** | 0.078 (±0.0023) | 0.011 | 66200 | 9360 | 0.022 (±0.0016) | 0.003 | 2240 | 317 | 0.066 | 0.009 |
| **44..*Gorilla*** | 0.243 (±0.0025) | 0.027 | 215000 | 23400 | 0.358 (±0.0018) | 0.039 | 59000 | 6440 | -0.005 | -0.001 |
| **43..*Pongo*** | 0.383 (±0.0028) | 0.022 | 242000 | 13700 | 0.359 (±0.0022) | 0.020 | 31600 | 1780 | 0.155 | 0.009 |
| **42..*Hylobates*** | 0.029 (±0.0024) | 0.001 | 6550 | 287 | -0.391 (±0.0018) | -0.017 | -8340 | -366 | 0.298 | 0.013 |
| **41..46** | 0.006 (±0.0015) | 0.001 | 454 | 82.8 | -0.054 (±0.0026) | -0.010 | -935 | -170 | 0.045 | 0.008 |
| **46..47** | 0.278 (±0.0037) | 0.045 | 29200 | 4700 | 0.124 (±0.0019) | 0.020 | 2310 | 371 | 0.200 | 0.032 |
| **47..48** | 0.084 (±0.0024) | 0.063 | 13300 | 98.9 | 0.066 (±0.0012) | 0.049 | 1530 | 1150 | 0.042 | 0.031 |
| **48..*Macaca*** | 0.093 (±0.0023) | 0.011 | 17900 | 2120 | -0.144 (±0.0015) | -0.017 | -3070 | -364 | 0.182 | 0.022 |
| **48..49** | 0.074 (±0.0022) | 0.150 | 13900 | 28300 | 0.031 (±0.0008) | 0.064 | 814 | 1660 | 0.041 | 0.084 |
| **49..*Papio*** | 0.354 (±0.0023) | 0.045 | 112000 | 14100 | 0.330 (±0.0015) | 0.042 | 13300 | 1680 | 0.130 | 0.016 |
| **49..50** | 0.073 (±0.0025) | 0.031 | 16200 | 6950 | 0.060 (±0.0014) | 0.026 | 1750 | 749 | 0.028 | 0.012 |
| **50..*Mandrillus*** | 0.231 (±0.0022) | 0.041 | 73800 | 13200 | 0.377 (±0.0015) | 0.067 | 18600 | 3330 | -0.022 | -0.004 |
| **50..*Cercocebus*** | -0.005 (±0.0022) | -0.001 | -1190 | -212 | -0.230 (±0.0015) | -0.041 | -5530 | -986 | 0.158 | 0.028 |
| **47..51** | 0.044 (±0.0022) | 0.024 | 6580 | 3590 | -0.059 (±0.0014) | -0.032 | -1180 | -644 | 0.078 | 0.043 |
| **51..*Cercopithecus*** | 0.029 (±0.0025) | 0.024 | 4770 | 601 | -0.228 (±0.0024) | -0.029 | -3330 | -420 | 0.185 | 0.023 |
| **51..*Erythrocebus*** | 0..198 (±0.0024) | 0.004 | 39600 | 4990 | -0.019 (±0.0016) | -0.002 | -352 | -44.4 | 0.211 | 0.027 |
| **46..52** | 0.230 (±0.0024) | 0.025 | 22800 | 4140 | 0.082 (±0.0020) | 0.015 | 1450 | 264 | 0.174 | 0.032 |
| **52..*Colobus*** | 0.149 (±0.0037) | 0.042 | 22600 | 2160 | -0.083 (±0.0019) | -0.008 | -1471 | -140 | 0.206 | 0.020 |
| **52..53** | 0.230 (±0.0026) | 0.014 | 14800 | 3670 | 0.097 (±0.0018) | 0.024 | 2130 | 530 | 0.036 | 0.009 |
| **53..*Presbytis*** | 0.149 (±0.0028) | 0.026 | 49300 | 7620 | 0.303 (±0.0017) | 0.047 | 10720 | 1660 | 0.024 | 0.004 |
| **53..*Pygathrix*** | 0.103 (±0.0024) | 0.036 | 6880 | 106 | -0.150 (±0.0017) | -0.023 | -3100 | -479 | 0.143 | 0.022 |
| **40..54** | 0.231 (±0.0024 | 0.006 | 900 | 40.8 | -0.576 (±0.0020) | -0.026 | -2710 | -123 | 0.431 | 0.020 |
| **54..55** | 0.041 (±0.0029) | 0.002 | 18300 | 2470 | 0.489 (±0.0021) | 0.066 | 2040 | 276 | 0.127 | 0.017 |
| **55..*Alouatta*** | 0.270 (±0.0026 | 0.020 | 24100 | 1800 | 0.327 (±0.0020) | 0.024 | 3380 | 252 | 0.047 | 0.004 |
| **55..56** | 0.225 (±0.0026) | 0.082 | 19000 | 6930 | 0.038 (±0.0017) | 0.014 | 276 | 101 | 0.199 | 0.073 |
| **56..*Lagothrix*** | 0.333 (±0.0026) | 0.031 | 54100 | 5080 | 0.198 (±0.0020) | 0.019 | 1910 | 179 | 0.198 | 0.019 |
| **56..*Ateles*** | 0.362 (±0.0026) | 0.034 | 61100 | 5730 | 0.385 (±0.0020) | 0.036 | 4710 | 441 | 0.099 | 0.009 |
| **54..64** | 0.159 (±0.0028) | 0.030 | 4250 | 794.7 | 0.033 (±0.0021) | 0.006 | 76.9 | 14.4 | 0.136 | 0.025 |
| **64..*Callicebus*** | 0.136 (±0.0027) | 0.009 | 5100 | 330 | -0.069 (±0.0022) | -0.004 | -155 | -10.1 | 0.183 | 0.012 |

| **BRANCH** | **Change in absolute brain mass** | | | | **Change in body mass** | | | | **Change in relative brain mass** | |
| --- | --- | --- | --- | --- | --- | --- | --- | --- | --- | --- |
|  | **Proportional change** | **Rate**  **(/million years)** | **Absolute change (mg)** | **Rate**  **(/million years)** | **Proportional change** | **Rate**  **(/million years)** | **Absolute change (mg)** | **Rate**  **(/million years)** | **Change** | **Rate**  **(/million years)** |
| **64..*Pithecia*** | 0.401 (±0.0027) | 0.026 | 21100 | 1370 | 0.153 (±0.0022) | 0.010 | 445 | 28.8 | 0.297 | 0.019 |
| **54..58** | 0.000 (±0.0015) | -0.000 | -5.87 | -2.26 | -0.045 (±0.0015) | -0.017 | -95.9 | -36.9 | 0.030 | 0.012 |
| **58..*Aotus*** | 0.249 (±0.0025) | 0.014 | 7460 | 410 | -0.027 (±0.0016) | -0.001 | -149 | -8.16 | 0.267 | 0.015 |
| **58..60** | 0.227 (±0.0031) | 0.087 | 6630 | 2550 | 0.053 (±0.0016) | 0.020 | 114 | 43.8 | 0.191 | 0.074 |
| **60..*Cebus*** | 0.640 (±0.0027) | 0.041 | 54700 | 3510 | 0.0493 (±0.0020) | 0.032 | 2100 | 135 | 0.303 | 0.019 |
| **60..*Saimiri*** | 0.269 (±0.0027) | 0.011 | 7730 | 495 | -0.179 (±0.0020) | -0.011 | -336 | -21.6 | 0.291 | 0.019 |
| **58..61** | -0.072 (±0.0030) | -0.013 | -1470 | -272 | -0.186 (±0.0020) | -0.034 | -308 | 57.0 | 0.263 | 0.049 |
| **61..*Saguinus*** | 0.088 (±0.0026) | 0.007 | 1830 | 143 | -0.180 (±0.0018) | -0.014 | -195 | -15.2 | 0.211 | 0.016 |
| **61..62** | -0.004 (±0.0024) | -0.003 | -72.4 | -49.9 | -0.028 (±0.0013) | -0.020 | -36.3 | -25.0 | 0.015 | 0.011 |
| **62..*Leontopithecus*** | 0.219 (±0.0025) | 0.019 | 5300 | 467 | 0.039 (±0.0018) | 0.003 | 51.3 | 4.52 | 0.192 | 0.017 |
| **62..63** | -0.013 (±0.0025) | -0.008 | -241 | -145 | -0.041 (±0.0013) | -0.025 | -48.8 | -29.4 | 0.015 | 0.009 |
| **63..*Callimico*** | 0.146 (±0.0024) | 0.015 | 3140 | 324 | -0.009 (±0.0018) | -0.001 | -9.97 | -1.03 | 0.152 | 0.016 |
| **63..*Callithrix*** | -0.015 (±0.0024) | -0.002 | -260 | -26.8 | -0.243 (±0.0018) | -0.025 | -210 | -21.7 | 0.152 | 0.016 |
| **39..40** | 0.919 (±0.0024) | 0.038 | 7700 | 316 | 0.900 (±0.0027) | 0.037 | 3560 | 146 | 0.303 | 0.012 |
| **39..*Tarsius*** | 0.533 (±0.0047) | 0.008 | 2550 | 38.0 | -0.570 (±0.0021) | -0.008 | -5.67 | -0.084 | 0.917 | 0.014 |
| **38..39** | 0.942 (±0.0220) | 0.091 | 927 | 90.0 | 0.976 (±0.0021) | 0.095 | 109 | 10.6 | 0.282 | 0.027 |
| **38..65** | 1.310 (±0.0218) | 0.064 | 2330 | 114 | 1.449 (±0.0018) | 0.071 | 1330 | 65.3 | 0.319 | 0.016 |
| **65..66** | 0.111 (±0.0050) | 0.004 | 713 | 26.6 | -0.377 (±0.0033) | -0.014 | -791 | -29.5 | 0.374 | 0.014 |
| **66..*Galago*** | 0.180 (±0.0034) | 0.006 | 1630 | 53.9 | -0.494 (±0.0029) | -0.017 | -402 | -13.3 | 0.514 | 0.017 |
| **66..67** | 0.224 (±0.0036) | 0.016 | 2140 | 149 | -0.033 (±0.0032) | -0.003 | -48.1 | -3.36 | 0.241 | 0.017 |
| **67..*Loris*** | 0.095 (±0.0031) | 0.006 | 1290 | 81.2 | -0.222 (±0.0028) | -0.014 | -218 | -13.7 | 0.249 | 0.016 |
| **67..*Nycticebus*** | 0.372 (±0.0031) | 0.023 | 7190 | 451 | 0.173 (±0.0028) | 0.011 | 259 | 16.3 | 0.256 | 0.016 |
| **65..68** | 0.226 (±0.0057) | 0.034 | 1680 | 250 | 0.006 (±0.0028) | 0.001 | 32.2 | 4.79 | 0.219 | 0.033 |
| **68..*Daubentonia*** | 1.039 (±0.0044) | 0.021 | 41000 | 814 | 0.301 (±0.0026) | 0.006 | 1390 | 27.5 | 0.837 | 0.017 |
| **68..69** | 0.120 (±0.0041) | 0.006 | 1320 | 61.7 | -0.119 (±0.0035) | -0.006 | -363 | -17.1 | 0.211 | 0.010 |
| **69..70** | -0.005 (±0.0026) | -0.003 | -56.3 | -34.1 | -0.034 (±0.0033) | -0.018 | -69.9 | -42.4 | 0.016 | 0.010 |
| **70..*Propithecus*** | 0.695 (±0.0034) | 0.025 | 21300 | 778.0 | 0.548 (±0.0023) | 0.020 | 2500 | 91.4 | 0.318 | 0.012 |
| **70..71** | -0.097 (±0.0029) | -0.030 | -1080 | -333 | -0.128 (±0.0032) | -0.040 | -253 | -78.3 | -0.008 | -0.002 |
| **71..*Lepilemur*** | 0.246 (±0.0032) | 0.010 | 3280 | 136 | 0.096 (±0.0023) | 0.004 | 190 | 2.89 | 0.176 | 0.007 |
| **71..72** | -0.112 (±0.0032) | -0.017 | -980 | -148 | -0.292 (±0.0033) | -0.044 | -353 | -53.1 | 0.086 | 0.013 |
| **72..*Cheirogaleus*** | 0.309 (±0.0030) | 0.018 | 3460 | 198 | 0.082 (±0.0023) | 0.005 | 78.5 | 4.48 | 0.252 | 0.014 |
| **72..*Microcebus*** | -0.273 (±0.0030) | -0.016 | -1560 | -88.9 | -0.841 (±0.0023) | -0.048 | -318 | -18.1 | 0.300 | 0.017 |
| **69..73** | 0.347 (±0.0036) | 0.031 | 6670 | 595 | 0.155 (±0.0033) | 0.014 | 466 | 41.6 | 0.238 | 0.021 |
| **73..*Varecia*** | 0.415 (±0.0033) | 0.023 | 19400 | 1090 | 0.294 (±0.0025) | 0.017 | 1490 | 83.4 | 0.212 | 0.012 |
| **73..*Eulemur*** | 0.284 (±0.0033) | 0.016 | 11200 | 627 | -0.037 (±0.0025) | -0.002 | -114 | -6.37 | 0.307 | 0.017 |

^1^ Nodal values were estimated in BayesTraits using a directional constant-variance random walk model for absolute brain mass, and a non-directional random walk model for body mass, relative brain sizes were inferred from these values as residuals on the phylogenetically controlled GLS regression equation (the ‘residuals second’ method: see table S3 and Methods). The proportional change is shown as the difference between the mean for each node with the 95% confidence intervals calculated from a distribution of changes between nodes produced by subtracting consecutive nodes for each run sampled. The rate of change along a branch was then calculated as the difference between the mean of the Bayesian MCMC nodal value estimates per unit time. Nodes refer to figure 2.

**6. Additional analyses in relation to *H. floresiensis***

The results of our main analyses are presented in the main body of the paper. Below are details of additional analyses. Table S5 shows the results of an analysis where we used the brain/body mass scaling relationships for each of the 10 branches which show a decrease in brain mass to produce a range of estimates for the expected decrease in brain mass given the estimated change in body mass from four possible ancestral populations and three possible body mass estimates for *H. floresiensis*.

**Table S5: Range of estimated decreases in brain mass during the evolution of *H. floresiensis* given scaling relationships during episodes of brain mass reduction**^1^

|  |  | **estimated range of decreases in brain mass given observed decrease in body mass** | |  |
| --- | --- | --- | --- | --- |
| **Ancestor** | ***H. floresiensis* body mass (kg)** | **minimum** | **maximum** | **actual calculated decrease in brain mass** |
| *H. erectus* | **16*** | -0.003 | -0.410 | -0.398 |
|  | 24 | -0.002 | -0.279 | -0.398 |
|  | 32 | -0.001 | -0.186 | -0.398 |
| Ngandong | **16*** | -0.003 | -0.426 | -0.450 |
|  | 24 | -0.002 | -0.296 | -0.450 |
|  | 32 | -0.002 | -0.203 | -0.450 |
| Dmanisi | **16*** | -0.003 | -0.368 | -0.216 |
|  | **24*** | -0.002 | -0.237 | -0.216 |
|  | 32 | -0.001 | -0.144 | -0.216 |
| *H. habilis* | **16*** | -0.002 | -0.243 | -0.137 |
|  | 24 | -0.001 | -0.112 | -0.137 |
|  | 32 | 0.000 | -0.020 | -0.137 |

^1^ Where the actual calculated decrease is within the range of estimated decreases the *H. floresiensis* body mass is highlighted in bold with an asterisk.

Next we asked whether the size of change in brain mass during the evolution of *Homo floresiensis* falls within the range observed elsewhere in the phylogeny using the two topologies proposed by Argue *et al.* [43]. The topology of hominins presented in figure 1 a were exchanged for the topologies of the two most parsimonious trees obtained by Argue *et al.* the topologies of the *Homini* are shown in Figure 1 b & c. We reconstructed the ancestral body and brain mass (see methods) for the node at which *H. floresiensis* splits from the rest of the tree. The analysis was run for each of the two most parsimonious trees separately and then for both trees together, taking advantage of BayesTraits ability to take phylogenetic uncertainty into account. The estimated values for the node at the base of the *H. floresiensis* terminal branch are shown in Table S6. These were then used to calculate an estimate for the change in brain mass along that branch in the same way as described for Table 2 (presented in Table 3) and Table S5 (presented in Table S7).

**Table S6: Estimated Log(body) and Log(brain) masses for the node at the base of the *H. floresiensis* terminal branch using the topologies proposed by Argue *et al.* [43]** ^1^

|  |  | **Estimates** | | | | |
| --- | --- | --- | --- | --- | --- | --- |
|  | ***H. floresiensis* body mass (kg)** | **Log(Body mass)** | | **Log(Brain mass)** | | **Relative brain size** |
| **Tree 1** | 16 | 4.633 | (±0.001) | 5.752 | (±0.002) | 0.404 |
|  | 24 | 4.649 | (±0.001) |  |  | 0.394 |
|  | 32 | 4.659 | (±0.001) |  |  | 0.387 |
| **Tree 2** | 16 | 4.611 | (±0.001) | 5.754 | (±0.003) | 0.422 |
|  | 24 | 4.625 | (±0.001) |  |  | 0.412 |
|  | 32 | 4.635 | (±0.001) |  |  | 0.405 |
| **Both trees** | 16 | 4.619 | (±0.001) | 5.754 | (±0.003) | 0.416 |
|  | 24 | 4.636 | (±0.001) |  |  | 0.404 |
|  | 32 | 4.647 | (±0.001) |  |  | 0.400 |

**^1^** Estimates are given as the mean with 95% confidence intervals.

**Table S7: Range of estimated decreases in brain mass during the evolution of *H. floresiensis* using the topologies proposed by Argue *et al.* [43] and given scaling relationships during brain mass reduction in primates^1^**

|  |  | **estimated range of decreases in brain mass given observed decrease in body mass** | |  |
| --- | --- | --- | --- | --- |
| **Ancestor** | ***H. floresiensis* body mass (kg)** | **minimum** | **maximum** | **actual calculated decrease in brain mass** |
| Argue Tree 1 | **16*** | -0.003 | -0.319 | -0.171 |
|  | **24*** | -0.002 | -0.199 | -0.171 |
|  | 32 | -0.001 | -0.114 | -0.171 |
| Argue Tree 2 | **16*** | -0.002 | -0.302 | -0.173 |
|  | **24*** | -0.001 | -0.182 | -0.173 |
|  | 32 | -0.001 | -0.097 | -0.173 |
| Both trees | **16*** | -0.002 | -0.308 | -0.173 |
|  | **24*** | -0.002 | -0.190 | -0.173 |
|  | 32 | -0.001 | -0.105 | -0.173 |

^1^ Where the actual calculated decrease is within the range of estimated decreases the *H. floresiensis* body mass is highlighted in bold with an asterisk.

Finally we used our best-fitting evolutionary model of brain evolution (directional) to estimate the expected brain size for *H. floresiensis* under a number of phylogenetic scenarios. This analysis thus attempts to predict the brain size of this species given the evolutionary model. This predicted brain size value can then be compared to the observed value. One significant problem with this approach is that it is currently not possible to analyse two correlated traits simultaneously (brain size and body size in this case) that evolved according different evolutionary models (directional model for brain size and non-directional model for body size, in this case). It is therefore not possible to incorporate body mass information into this analysis. Hence, given the strong directional component to brain evolution it is unlikely that the model will estimate a decrease in brain mass along a terminal branch where the tip value is not known, because information on changes in encephalization caused by the evolution of body mass will be lost. For example we performed this analysis for the three species (*Microcebus*, *Callithrix,* *Cercocebus*) where the terminal branch shows a decrease in brain mass are overestimated by 3.8-7.4% (Table S2: labelled in red). To our knowledge no model has been developed which can incorporate co-evolution between traits which evolve under different modes, the ability to do so would no doubt be a useful advance in comparative methods. However comparing the predicted values under multiple scenarios may still indicate which fits most closely to the observed *H. floresiensis* brain mass. We estimated the Log(brain mass) for *H. floresiensis* setting *H. floresiensis* in turn as a sister taxa to *H. erectus*, Dmanisi, Ngandong hominoids and *H. habilis*, to test the insular dwarfism model, and under the topologies proposed by Argue *et al.* [43]. Under the insular dwarfism model the terminal branches of *H. floresiensis* and the sister species was set as 190k years, the age of the oldest hominin artefacts found in Liang Bua [27]. The results are presented in table S8, for comparison we estimated the expected value of the other hominins in the phylogeny shown in Figure 1, on average estimates were within 1.84% of the real value, with a range between 0.74-3.47% (data not shown).

**Table S8: Predicted Log(brain mass) of *H. floresiensis* under a number of phylogenetic scenarios**^1^

| **a) Insular dwarfism** | **Ancestor** | **Estimated *H. floresiensis* Log(brain mass)** | | **% error** |
| --- | --- | --- | --- | --- |
|  | *H. erectus* | 5.968 | (±0.002) | 6.943 |
|  | Ngandong | 6.003 | (±0.002) | 7.576 |
|  | Dmanisi | 5.846 | (±0.002) | 4.762 |
|  | *H. habilis* | 5.764 | (±0.002) | 3.283 |
|  |  |  |  |  |
|  |  |  |  |  |
| **b) Argue *et al.* (2009)** | **Phylogeny** | **Estimated *H. floresiensis* Log(brain mass)** | | **% error** |
|  | Tree 1 | 5.868 | (±0.002) | 4.890 |
|  | Tree 2 | 5.848 | (±0.004) | 4.567 |
|  | Both trees | 5.859 | (±0.004) | 4.745 |

**^1^** Estimates are given as the mean with the 95% confidence intervals.

Estimates made under all scenarios are larger than the real log brain mass (mg) of 5.581 as expected. It is notable that the estimates for descent from a *H. habilis* or Dmanisi ancestor have the lowest percentage errors, which is consistent with the results of our other analyses. The error for a shared ancestry with *H. habilis* also falls within the range seen when estimating other hominins, whilst the error associated with a descent from Dmanisi hominids or under Argue *et al.*’s proposed typologies are also reasonably low.
